# Effect of Melatonin Supplementation on In Vitro Developmental Competence of Bovine Oocyte: A Systematic Review and Meta-Analysis

**DOI:** 10.1155/vmi/5351950

**Published:** 2025-10-24

**Authors:** Najmeh Davoodian, Ali Kadivar, Aziz A. Fallah, Nahid Davoodian

**Affiliations:** ^1^Research Institute of Animal Embryo Technology, Shahrekord University, Shahrekord, Iran; ^2^Department of Clinical Sciences, Faculty of Veterinary Medicine, Shahrekord University, Shahrekord, Iran; ^3^Department of Food Hygiene and Quality Control, Faculty of Veterinary Medicine, Shahrekord University, Shahrekord, Iran; ^4^Department of Clinical Biochemistry, Faculty of Medicine, Hormozgan University of Medical Sciences, Bandar Abbas, Iran; ^5^Endocrinology and Metabolism Research Center, Hormozgan University of Medical Sciences, Bandar Abbas, Iran

**Keywords:** cattle, embryo, melatonin, meta-analysis, oocyte

## Abstract

Melatonin application in in vitro embryo production (IVEP) constitutes a promising research focus. Nevertheless, there remains a lack of comprehensive meta-analytical evidence assessing its effects in the bovine species. The present systematic review and meta-analysis study performed an in-depth overview of the effects of melatonin in different steps of the IVEP in cattle to provide insights into its application. A literature search on three major databases identified related articles until 3 December 2024. The overall effect was calculated as odds ratio (OR) and 95% confidence interval (CI) in the random effects model. Meta-analysis revealed that melatonin in maturation media significantly increased nuclear maturation rate [OR = 1.33 (1.11, 1.60), *p*=0.002], cleavage rate [OR = 1.26 (1.11, 1.43), *p* ≤ 0.001], and blastocyst rate [OR = 1.27 (1.14, 1.43), *p* ≤ 0.001], while it did not affect the hatching rate of blastocysts. Subgroup analysis based on the concentration of melatonin demonstrated that low concentration was superior in effects. Melatonin in culture media did not affect the odds of cleaved embryos, and blastocysts, however, increased the rate of hatching [OR = 1.35 (1.04, 1.76), *p*=0.025]. Subgroup analysis showed the significant effect of low and moderate concentrations on the odds of blastocysts and hatched blastocysts. No publication bias was detected. Descriptive synthesis of data suggested improvements in maturation, developmental quality, fertilization ability, and epigenetic modification during maturation and enhancement in the quality of embryos through modifications in transcription in blastocysts to alleviate apoptosis during culture. This review concludes that the application of melatonin is more promising in maturation media than in culture.

## 1. Introduction

As an assisted reproductive technique, the in vitro embryo production (IVEP) to improve the number of offspring from superior animals has been noticed extensively. Moreover, the influence of some reproductive disorders, diseases, and environmental stresses can be eliminated using this method. Over the past few decades, IVEP of bovine embryos has gained significant popularity. Obviously, the commercial application and request for these embryos are growing fast with an average growth rate of 12% annually [1]; however, this technique faces challenges such as inefficiency, which is partly due to the difficulty in replicating the natural processes of oocyte maturation, fertilization, and embryo development in vitro. During the process of IVEP, various external environmental factors, e.g., exposure to nonoptimal conditions of oxygen, temperature, pH, and light, significantly contribute to the excessive accumulation of reactive oxygen species (ROS), leading to oxidative stress (OS), which hinders the optimal development of these embryos. Free radicals are produced normally in the metabolic pathways, playing the role of physiological second messengers in cellular functions [2]; however, they are highly reactive with complex molecules, e.g., proteins, lipids, and DNA in cells, leading to serious damage and even cell death [3]; the oocytes of domestic animals are particularly susceptible to such events as they are rich in lipid content in the cytoplasm. To enhance oocyte and embryo development, additional culture supplements, such as antioxidants, are being explored to help manage ROS; in this context, melatonin has been introduced to improve the development of embryos [4].

In mammals, melatonin (N-acetyl-5-methoxy tryptamine) from the pineal gland transmits the signals of photoperiodic information to the hypothalamic–pituitary–gonadal axis to affect the sexual maturation and reproductive function [5]. Since its discovery, melatonin has been introduced as an anti-inflammatory and antioxidant agent with extensive application in the fields of human and veterinary medicine. In this contest, the animal reproduction industry has been an area for the application of this compound as an antioxidant to scavenge the detrimental effects of free radicals on gametes and embryos. The membrane and nuclear receptors of melatonin have been identified in mammalian ovaries [6]; the synthesis of melatonin in bovine cumulus–oocyte complexes (COCs) [7] and FF has been demonstrated; and a correlation has been shown between melatonin and the health and development of preovulatory follicles [6, 8]. Opposite the many other free-radical scavengers, melatonin is a multifunctional molecule with universal distribution [9] affecting indirectly the stimulation and inhibition of antioxidative and pro-oxidative enzymes.

The role of melatonin in livestock reproduction is well investigated [10]. Melatonin supplementation has been extensively applied not only in vivo to enhance reproductive outcomes in cattle [11, 12], sheep [13, 14], goats [15], and buffaloes [16] but also in assisted reproduction [17, 18]. The role of melatonin in the development of oocytes from different animal species such as cattle [7, 19, 20], pigs [21–23], buffaloes [24–27], sheep [28, 29], and camels [30] has been well demonstrated. Moreover, different mechanisms are introduced that are responsible for the action of melatonin in promoting the development of bovine oocytes and embryos including receptor signal transduction, antioxidative, and antiapoptotic mechanisms [31–34].

The application of melatonin supplement in different steps of the IVEP process in cattle has been noticed extensively; however, it has brought various results and yielded diverse outcomes; this variety is obvious even in the determination of the most effective concentration of melatonin in media. Notwithstanding those numerous studies that evaluated the effect of melatonin supplements on different aspects of bovine oocyte development with controversial results, there is no study to enable us to decide on the application of this supplement in commercial IVEP in cattle. Therefore, the present systematic review and meta-analysis study aimed to perform an in-depth overview of the effects of melatonin in different steps of the IVEP process in cattle to provide insights into the best stage for its application in improving efficiency. Our objectives are to identify the stage of IVEP where melatonin supplementation yields the greatest improvement in efficiency and to determine the optimal concentration of this antioxidant in culture media for enhancing IVEP outcomes.

## 2. Methods

This systematic review and meta-analysis were conducted in accordance with the Preferred Reporting Items for Systematic Reviews and Meta-Analyses (PRISMA) guidelines. The PRISMA checklist was used to ensure comprehensive and transparent reporting.

### 2.1. Strategies of the Search

Three major scientific databases, Web of Science (WOS), Scopus, and PubMed, were searched in titles, abstracts, and keywords to find related articles on December 3, 2024. Keywords “cattle OR cow OR bovine OR zebu OR yak OR bos indicus OR taurus OR Holstein OR dairy OR beef” AND “Melatonin AND in vitro AND oocyte” AND “embryo OR development OR maturation OR meiosis OR blastocysts OR preimplantation OR M phase OR embryo” were applied in databases. The details are provided in Supporting 1.

### 2.2. Eligibility of Studies

The criteria for eligibility were determined in the PICO based on Page et al. 2021 [35]:

- P (population): Including female cattle (dairy, beef, and heifer) who were subjected to oocyte recovery from their ovaries (using ovum pickup (OPU) or after slaughter).
- I (intervention): The application of a melatonin supplement in media to develop the oocytes into embryos in a standard in vitro model without the induction of any artificial stress.
- C (comparison): The comparison of the control group, which received no treatment.
- O (outcomes): Defined in three important parts of an IVEP process including the maturation of oocytes, the ability to be fertilized, and the development of embryos up to hatched blastocysts.

#### 2.2.1. Inclusion and Exclusion Criteria

##### 2.2.1.1. Intervention and Control

In this review, only those studies performed on the bovine oocytes included in an IVEP program were selected in which a medium was supplemented with melatonin at any concentration. Any modification of melatonin was removed. Any experiment performed in the presence of abnormal conditions to induce osmotic and temperature stress was removed. The oocytes that received no treatment under the same conditions were determined as the control group.

##### 2.2.1.2. Outcomes of Interest

In this review, we analyzed the data related to nuclear and cytoplasmic maturation outcomes, as well as cleavage and blastocyst rates (BRs), alongside the quality of embryos derived from oocytes or zygotes that were cultured in supplemented media. Any experiment in abnormal conditions of culture, e.g., heat stress, oxygen tension, or any chemical intervention, was removed. Moreover, the data of melatonin receptor evaluation, parthenogenetic or cloned embryos, specific media composition, any treatment on the sperm applied for fertilization, protein assays, epigenetics, RNA-Seq, metabolites in spent media, and genetic tests were excluded from the study.

#### 2.2.2. Selection of Studies and Extraction of Data

Two independent reviewers evaluated the search results separately based on the defined PICO to exclude irrelevant articles, determine the right articles, and then extract the relevant data including the first author's name, year of publication, the dosage and duration of treatment, justification of the study, main outcomes of interest, and excluded data. The GetData Graph Digitizer software version 2.24 was used for the extraction of data from those articles that presented the outcome variables only in graphic format.

### 2.3. Statistical Analysis

This study employed the Stata software Version 11.2 (Stata Corporation, College Station, TX, United States) to perform statistical analyses. The effect sizes were calculated from discontinuous outcomes of intervention versus control groups and presented as odds ratio (OR) with 95% confidence interval (CI) using a random effects model to calculate the overall effect [36]. Melatonin concentration in the media was considered for subgroup analyses. The results were displayed on forest plots. The interstudy heterogeneity was determined using the Cochrane's *Q* test, quantified by the I-squared (*I*^2^) statistic, ranging from 0% to 100% in which *I*^2^ > 50% refers to considerable heterogeneity [37]. The Begg and Mazumdar adjusted rank correlation test [38] and the Egger regression asymmetry test [39] were employed to assess the potential publication bias. *p* < 0.050 is considered statistically significant.

## 3. Results

### 3.1. The Identification and Selection of Studies

**Figure 1:**
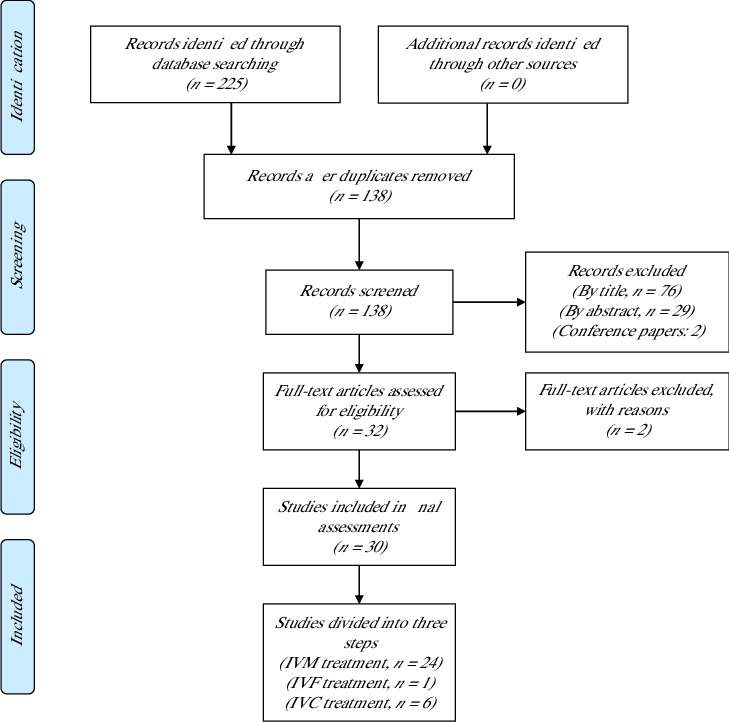
PRISMA flow diagram of the study.

The initial search yielded 225 results after which the eligibility criteria were applied to narrow the results to 30 articles. The details are provided in Figure 1. In the first stage, the articles were evaluated to determine whose titles did not fall within the scope of this study after removing 87 duplicates. The remaining articles (*n* = 138) were screened to exclude two conference papers and 76 articles by title and then subjected to the study of the abstract, leading to the exclusion of 29 articles. The remaining 32 articles were screened out to remove one Portuguese article. Moreover, the authors did not have access to one article even upon request from the corresponding author, and finally, 30 articles were subjected to data extraction. All these articles are justified as they have provided data related to evaluating the effect of melatonin in the maturation or fertilization or culture media on the rate of maturation, and/or fertilization, and/or development of bovine oocytes and embryos.

### 3.2. The Characteristics of Studies

The details for the main characteristics of the 30 articles are provided in Table 1. In short, 24 articles evaluated the melatonin supplement in maturation media [7, 19, 20, 32, 34, 40–58], 1 article in fertilization media [59], and 6 articles in culture media [19, 33, 60–63]; one of the articles evaluated the treatment in both maturation and culture media [19]. Table 1 includes information on the number of bovine oocytes in experiments, the outcomes of maturation, cleavage and BRs, the quality assessment of oocytes and embryos, and also all the data in the article that did not relate to the scope of this review. The dosage of the melatonin supplement was in a wide range between 1 mM and 0.1 pM.

To systematically evaluate, the results of meta-analyses and descriptive synthesis of data were divided into three sections, as follows.

### 3.3. Section 1—Melatonin as a Supplement to Maturation Media

#### 3.3.1. Meta-Analysis

In this review, a total of 30,935 oocytes, which were collected from different animals, were included (21,909 in the treatment group and 9026 in the control group) to evaluate the effect of melatonin supplement in maturation media on a wide range of parameters.

**Figure 2:**
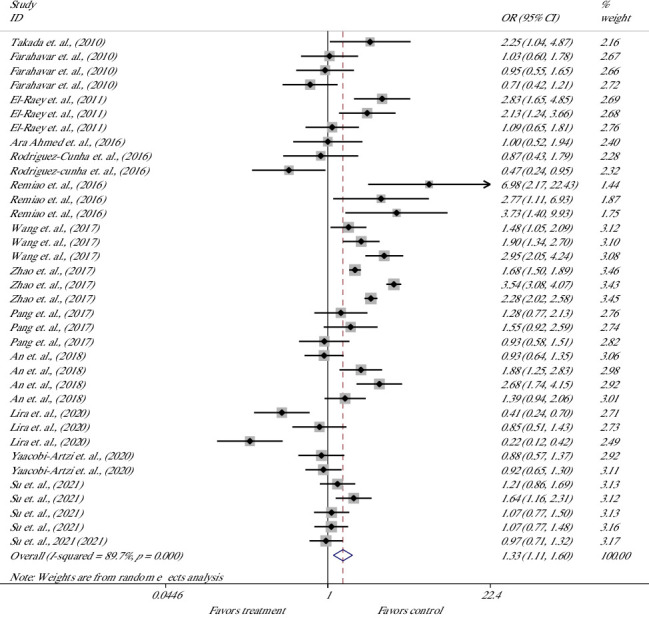
Forest plot of the effect of melatonin supplement in maturation media on the nuclear maturation of bovine oocytes.

In bovine oocytes, the effect of melatonin treatment on nuclear maturation (NM) was evaluated in 36 studies of 12 articles [7, 20, 32, 40, 41, 45, 47–50, 53, 55, 56] (Figure 2). Meta-analysis of 36 experiments suggested that melatonin supplementation significantly increased the rate of NM, indicating 33% higher odds of success with melatonin versus control; the overall OR was 1.33; the lower and upper limits of the 95% CI were 1.11 and 1.60, respectively [OR = 1.33 (1.11, 1.60), *p*=0.002]. The heterogeneity of included studies was considerable, with an *I*^2^ value of 89.7% and Cochrane's *Q* test (*p* ≤ 0.001).

**Figure 3:**
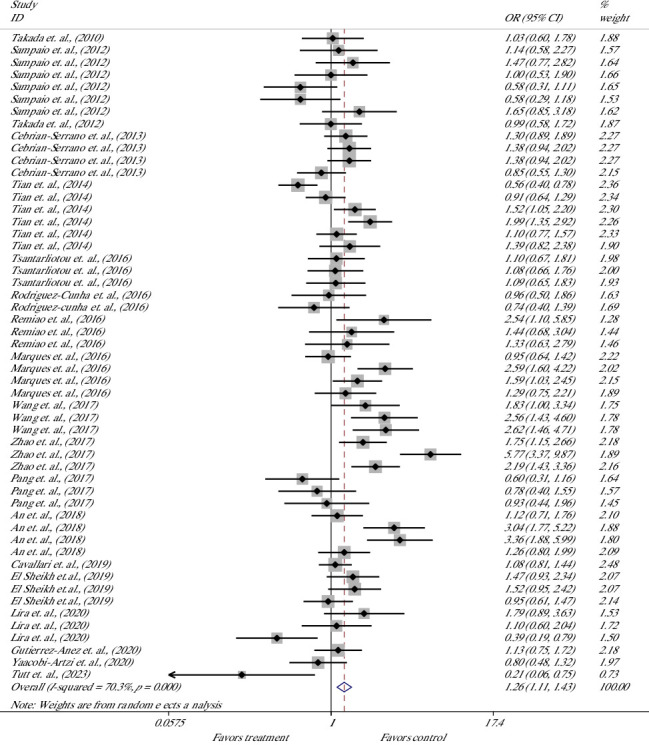
Forest plot of the effect of melatonin supplement in maturation media on the cleavage rate of bovine presumptive zygote.

The effect of melatonin on the cleavage rate (CR) of bovine presumptive zygotes was evaluated in 53 studies of 19 articles [19, 20, 32, 34, 40, 42–44, 46–55, 58] (Figure 3). Meta-analysis suggested that melatonin significantly increased the rate of cleavage, indicating 26% higher odds of success with melatonin versus control; the overall OR was 1.26; the lower and upper limits of the 95% CI were 1.11 and 1.43 [OR = 1.26 (1.11, 1.43), *p* ≤ 0.001]. The heterogeneity of included studies was considerable, with an *I*^2^ value of 70.3% and Cochrane's *Q* test (*p* ≤ 0.001).

**Figure 4:**
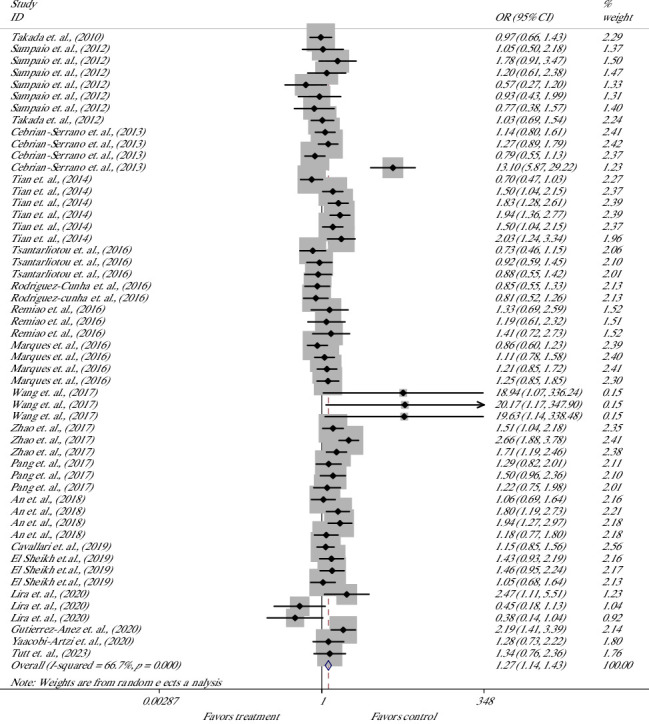
Forest plot of the effect of melatonin supplement in maturation media on the blastocyst rate derived from bovine oocytes.

The effect of melatonin treatment on the BR derived from bovine-treated COCs was evaluated in 53 studies of 19 articles [19, 20, 32, 34, 40, 42–44, 46–55, 58] (Figure 4). Meta-analysis suggested that melatonin significantly increased the chance for the formation of blastocysts, with 27% higher odds of success with melatonin versus control; the overall OR was 1.27; the and lower and upper limits of the 95% CI were 1.14 and 1.43, respectively [OR = 1.27 (1.14, 1.43), *p* ≤ 0.001]. The heterogeneity of included studies was considerable with an *I*^2^ value of 66.7% and Cochrane's *Q* test (*p* ≤ 0.001).

**Figure 5:**
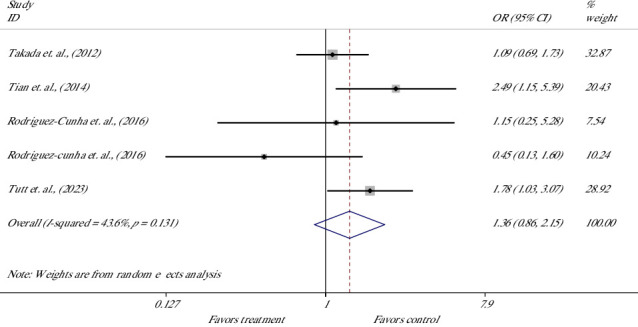
Forest plot of the effect of melatonin supplement in maturation media on the hatched-blastocyst rate.

The effect of treatment on the hatched BR (HBR) was evaluated in five studies of four articles [34, 43, 47, 58] (Figure 5). Meta-analysis suggested no significant difference in the chance for the hatching of blastocysts; OR was 1.36 with 95% CI between 0.86 and 2.15 [OR = 1.36 (0.86, 2.15), *p*=0.181]. The heterogeneity of included studies was not significant, with an *I*^2^ value of 43.6% and Cochrane's *Q* test (*p*=0.131).

#### 3.3.2. Subgroup Analysis

To determine the best concentration of melatonin in maturation media, the different dosages were categorized into low (1 nM–0.1 pM), moderate (10 μM–10 nM), and high (1 mM–0.1 mM). The studies were categorized into three groups and evaluated for NM, CR, BR, and HBR outcomes. The results of subgroup analysis are summarized in Table 2, which reports the OR with 95% CIs, *p* values for significance, and *I*^2^ values and corresponding *p* values for heterogeneity.

Low concentrations of melatonin significantly increased NM [OR = 1.52, 95% (1.16–1.99), *p* = 0.002] [20, 32, 40, 45, 47–50, 53, 56] and CR [OR = 1.42, 95% (1.20–1.69), *p* ≤ 0.001] [19, 20, 32, 34, 40, 42–44, 47–50, 52, 53], though both outcomes showed considerable heterogeneity (*I*^2^ = 90.8% and 68.3%, respectively; both *p* ≤ 0.001). High and moderate concentrations did not show a significant effect on these outcomes.

For BR, improvements were observed with both low concentration [OR = 1.35, 95% (1.18–1.55), *p* ≤ 0.001] [19, 20, 32, 34, 42–44, 47–50, 52, 53] and moderate concentration [OR = 1.20, 95% (1.03–1.40), *p*=0.019] [19, 20, 32, 34, 42, 46–48, 50–53, 55, 58, 64] again with notable heterogeneity (*I*^2^ = 54.9%, *p* ≤ 0.001; and *I*^2^ = 53.8%, *p* = 0.003, respectively). HBR was significantly improved only with moderate concentrations [OR = 1.69, 95% (1.01–2.83), *p*=0.045] [47, 58], with no observed heterogeneity (*I*^2^ = 0.0%, *p*=0.595).

#### 3.3.3. Publication Bias

The results of the Begg and Mazumdar adjusted rank correlation test [38] showed that there was no publication bias for NM rate, CR, BR, and HBR. Moreover, the results of Egger's regression asymmetry test [39] showed that there was a publication bias for NM rate but not for CR, BR, and HBR. The results of funnel plots are presented in Supporting 2. In brief, for NM (Supporting 2, Figure 1), the assessment of the Begg and Mazumdar adjusted rank correlation test showed symmetry (*p* = 0.870), whereas the Egger regression asymmetry test showed asymmetry (*p*=0.002). For CR (Supporting 2, Figure 2), the assessment of publication bias using the Begg and Mazumdar adjusted rank correlation test and the Egger regression asymmetry test showed symmetry; Begg's rank test (*p*=0.586) and Egger's regression test (*p*=0.992) indicated no significant evidence of publication bias. For BR (Supporting 2, Figure 3), the assessment of publication bias using the Begg and Mazumdar adjusted rank correlation test and the Egger regression asymmetry test showed symmetry; Begg's rank test (*p*=0.860) and Egger's regression test (*p*=0.439) indicated no significant evidence of publication bias. For HBR (Supporting 2, Figure 4), the assessment of publication bias using the Begg and Mazumdar adjusted rank correlation test and the Egger regression asymmetry test showed symmetry; Begg's rank test (*p*=1.00) and Egger's regression test (*p*=0.727) indicated no significant evidence of publication bias.

#### 3.3.4. The Descriptive Results

The parameters related to cytoplasmic maturation of oocytes and subsequent development as well as quality assessments and relative transcription are presented in Table 3. In cumulus cells (CCs) treated with melatonin supplementation during maturation, the diameter of COCs did not change [45]. Although one study reported improved expansion of the CC layer [7], other studies found no change [7, 41, 45, 55] or even a reduction in cumulus expansion [55]. The live cell percent did not change [48]. DNA damage was not observed [47] or was significantly reduced [43, 47] following treatment. Steroidogenic-related transcripts CYP11A1, CYP19A1, and StAR upregulated [49]; CC-specific transcripts PTX3, HAS1, HAS2, LHR1, LHR2, and EGFR upregulated, whereas TNFAIP6, GREM1, and PTX3 did not change [34, 57].

In oocytes treated with melatonin supplementation during maturation, treatment led to a significant reduction in spindle disorganization [50], higher peripheral and cortical distribution [20, 32], and improved normal distribution of endoplasmic reticulum (ER) [32]. The distribution of mitochondria [7, 32] and mitochondrial membrane potential (MMP) [20] was improved, whereas other studies reported no change in the activity of mitochondria [7] and MMP [19]. The higher levels of adenosine triphosphate (ATP) [32] and glutathione (GSH) [20, 32] and lower ROS [7, 20, 32, 47, 48, 50, 56] were reported, whereas there are reports on no change in the levels of ROS [19, 47, 51] and GSH [19, 48]. Melatonin did not change [20] the rate of apoptosis or reduce it [50]. Antioxidant-related transcripts catalase (CAT), superoxide dismutase 1 (SOD1), and glutathione peroxidase (GPX) upregulated, and Cu, ZnSOD, MnSOD, and GPX4 did not change [32, 47]. Development-related transcripts Tet1, Tet2, Tet3, GDF9, MARF1, and DNMT1a upregulated, whereas Dnmt1 downregulated [32, 34].

In embryos derived from melatonin-treated oocytes, treatment did not affect the rate of DNA damage in blastocysts based on the COMET assay [19, 40] and GSH and ROS levels [19], while there are reports on reduction in ROS [49], apoptosis [20, 48, 50], polyspermy and unfertilized oocytes, and improved pronucleus formation [32]. The proportion of morula and Grade 1 and 2 blastocysts did not change or decreased although there is a report on increased Grade 2 blastocysts [19]. TCN and ICM/TE increased [32, 34, 48, 50, 54] or were not affected by the treatment [19, 20, 34, 44, 50]. Antioxidant-related transcripts GPX, SOD1, SOD2, CAT, XIAP, and MCL1 upregulated [19, 20, 48]. Apoptosis-related transcript BCL-2 upregulated, whereas CASP3, BAX, and SHC1 downregulated [32, 48, 57]. Development-related transcript HSP70 upregulated, whereas OCT4, NANOG, PU5F1, SLC1A, SLC2A, SLC3A, HSPB1, and KRT8 did not change [19, 20, 48, 57].

### 3.4. Section 2—Melatonin as a Supplement of Fertilization Media

In a sole research study [59] to explore the effect of melatonin supplementation of fertilization media on the subsequent fertilization and development of mature bovine COCs, two categories of different melatonin concentrations were evaluated separately. The results showed that high concentrations of melatonin (0.01, 0.1, and 1 mmol) did not affect the CR other than reducing the BR in the 1 mM group. However, low concentrations of melatonin (10, 100, and 1000 nmol) did not affect the cleavage and BRs.

### 3.5. Section 3—Melatonin as a Supplement of Culture Media

#### 3.5.1. Meta-Analysis

In this review, a total of 4481 presumptive zygotes or blastocysts were included (5208 in the treatment group and 2273 in the control group) [19, 33, 60–63]; meta-analysis was performed to evaluate the parameters including the rates of cleavage, blastocyst, and HBRs as below.

**Figure 6:**
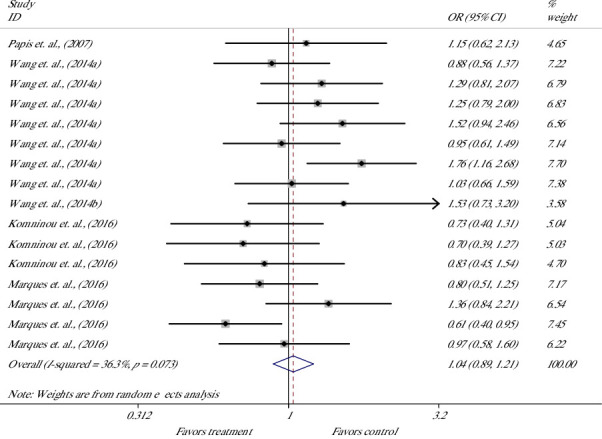
Forest plot of the effect of melatonin supplement in culture media on the cleavage rate of bovine presumptive zygote.

The effect of melatonin treatment in culture media on the CR of bovine presumptive zygotes was evaluated in 16 studies from 5 articles [19, 33, 60–62] (Figure 6). Meta-analysis showed no significant OR and suggested that melatonin supplement did not affect the chance for cleaved embryos [OR = 1.04 (0.89, 1.21), *p*=0.637; *I*^2^ = 36.3%, Cochrane's *Q* test (*p*=0.073)].

**Figure 7:**
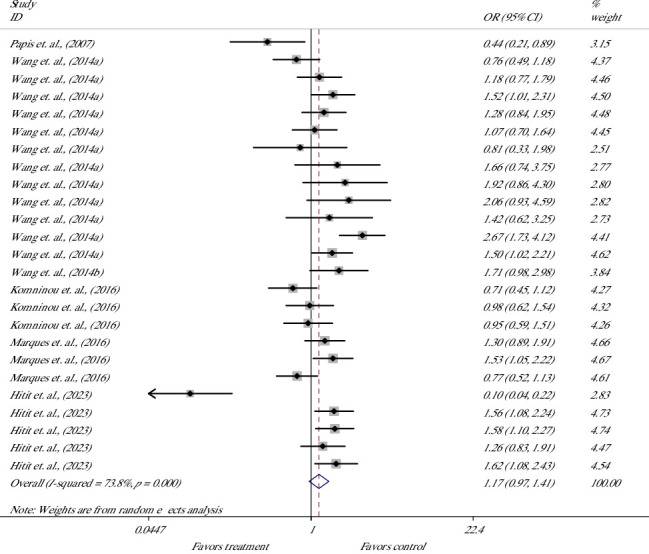
Forest plot of the effect of melatonin supplement in culture media on the blastocyst rate of bovine presumptive zygote.

The effect on the BR was evaluated in 25 studies from 6 articles [19, 33, 60–63] (Figure 7). Meta-analysis showed no significant OR and suggested that melatonin supplement had no effect on the chance for blastocysts [OR = 1.17 (0.97, 1.41), *p*=0.111; *I*^2^ = 73.8%, Cochrane's *Q* test (*p* ≤ 0.001)].

**Figure 8:**
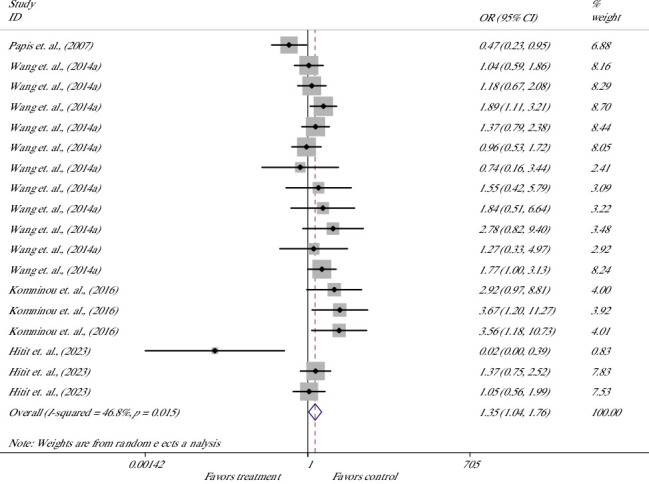
Forest plot of the effect of melatonin supplement in culture media on the hatched-blastocyst rate.

The effect on the hatching rate of blastocysts was evaluated in 18 studies from 4 articles [60–63] (Figure 8). Meta-analysis suggested that melatonin supplement had a 35% higher odds of success for hatching in the melatonin group versus the control group and significantly increased the rate of hatching; the lower and upper limits of the CI were 1.04 and 1.76, respectively [OR = 1.35 (1.04, 1.76), *p*=0.025]. The heterogeneity of included studies was moderate with an *I*^2^ value of 46.8% and Cochrane's *Q* test (*p*=0.015).

#### 3.5.2. Subgroup Analysis

The results of the subgroup analysis are presented in Table 4, which reports the OR with 95% CIs, *p* values for significance, and *I*^2^ values and corresponding *p* values for heterogeneity.

Low concentrations of melatonin did not show a significant effect on CR [19, 61, 62], but significantly increased BR [OR = 1.26, 95% (1.07–1.49), *p*=0.007] [19, 61–63] and HBR [OR = 1.54, 95% (1.12–2.14), *p*=0.009] [61–63], though both outcomes showed no heterogeneity (*p* > 0.05).

Moderate concentrations of melatonin did not show a significant effect on CR [19, 61, 62] but significantly increased BR [OR = 1.44, 95% (1.15–1.79), *p* ≤ 0.001] [19, 61–63] with notable heterogeneity (*I*^2^ = 54.4%, *p*=0.02) and HBR [OR = 1.57, 95% (1.17–2.11), *p*=0.003] [61–63] with no heterogeneity (*p* > 0.05).

High concentrations of melatonin did not show a significant effect on CR [60, 61], BR [60, 61, 63], and HBR [60, 61, 63] (*p* > 0.05).

#### 3.5.3. Publication Bias

The results of the Begg and Mazumdar adjusted rank correlation test showed that there was no publication bias for CR and HBR other than for BR. Moreover, based on the results of the Egger regression asymmetry test, no publication bias for CR, BR, and HBR was detected. The results are presented in Supporting 3. In brief, for CR (Supporting 3, Figure 1), the assessment of publication bias using funnel plots showed symmetry; Begg's rank test (*p*=0.787) and Egger's regression test (*p*=0.847) indicated no significant evidence of publication bias. For BR (Supporting 3, Figure 2), the funnel plot of Begg's rank test showed asymmetry (*p*=0.036), whereas the funnel plot of Egger's regression asymmetry test showed symmetry (*p*=0.162). For HBR (Supporting 3, Figure 3), funnel plots showed symmetry; Begg's rank test (*p*=0.472) and Egger's regression test (*p*=0.990) indicated no significant evidence of publication bias.

#### 3.5.4. The Descriptive Results

The results of descriptive outcomes are presented in Table 5. Qualitative assessment of embryos suggested an improvement in the development of Grade 1 embryos [19]. A decrease in the rate of blastomere apoptosis was observed following treatment [19, 62, 63]; however, caspase activity remained unchanged [63]. The levels of ROS [19] and GSH [19] in blastocysts did not change, but ROS decreased in 8-cell embryos in a sole reporting study [62]. TCN of blastocysts was reported to be increased [48, 61, 62] but mostly not affected by the treatment [19, 60, 61, 63]. Moreover, a sole study evaluating the quality of blastocysts based on the mortality and hatching rates after freeze–thaw reported significant increases and decreases in the hatching rate and mortality, respectively [33] (not included in the table).

## 4. Discussion

This systematic review evaluated 30 relevant assisted reproductive technique (ART) articles for the quantitative and qualitative analysis of outcomes to conclude the application of melatonin as a supplement for IVEP in cattle. Accumulating data indicated that melatonin in maturation media increased the likelihood of bovine oocyte NM [7, 20, 32, 41, 48–50, 53] alongside the improved cytoplasmic maturation [7, 19, 20, 32, 50], reduced ROS [7, 20, 32, 47, 48, 50, 56], and apoptosis [20, 50] and upregulation of some antioxidant enzyme-related genes [20, 32, 47, 48] and development-related genes [32, 34]; CCs were affected in transcription and reduced DNA damage [34, 43, 49, 57]. Melatonin exposure was associated with significantly higher odds of the cleaved embryos [19, 32, 34, 42, 48–50, 52] and blastocysts [20, 32, 34, 42, 48–50, 52, 53]; the hatching of blastocysts was not affected by the treatment, but the improved quality of blastocysts was represented by a lower apoptosis rate [20, 48, 50] alongside the downregulation of apoptosis-related genes [20, 48].

Melatonin in this study was determined to increase the chance for maturation by 33%; however, there is high heterogeneity that arises from the diversity in the number of oocytes subjected to experiments. Contrarily, a wide range of dosages was considered in different studies. Determining the best concentration of melatonin made direction for performing a subgroup meta-analysis to explore that the low concentrations of melatonin significantly increased the maturation rate compared to nontreated oocytes [20, 32, 48–50, 56]; high heterogeneity suggests that certain dosages in this category are more effective than others. In this regard, the toxicity of melatonin in meiotic maturation has been regarded as improbable [41]; however, we showed that moderate concentrations were not perfect enough. Publication bias was not detected other than NM through the Egger regression asymmetry test [39] but not the Begg and Mazumdar test [38].

Melatonin is demonstrated to enhance the meiotic maturation of porcine [22], buffalo [24], and mouse [65] oocytes in vitro through induction of meiotic resumption, but in bovine oocytes, it is mentioned to induce the progression from MI to MII stages specifically through counteracting the free-radical stressors in IVM medium [7] alongside the role of inducing the expression of steroidogenic genes (CYP11A1, CYP19A1, and StAR) to increase the secretion of progesterone and estradiol, which improve the oocyte maturation [49]. In this context, melatonin's role in modulating steroidogenesis and enhancing oocyte quality has been well-studied and documented [66]; steroid hormones initiate and finely regulate the maturation of oocytes both directly and via classical steroid receptor-mediated pathways, while also sustaining critical communication between oocytes and surrounding CCs [67, 68].

It is obvious that alongside NM, cytoplasmic maturation accounts for another important event essential to be completed before fertilization. This event involves the rearrangement of cytoplasmic organelles and the storage of proteins and transcripts necessary for the subsequent development of oocytes up to EGA [69]. The distribution of cortical granules (CGs) makes the oocytes ready for quick action against polyspermy [32]; mitochondria experience changes in abundance, activity, localization, and membrane potential to meet the demands for energy in maturing oocytes [7, 70]; and the polarity of the mitochondrial membrane is essential for the proper action of respiratory complexes and ATP synthesis [71]. The decrease in the MMP induces the early phase of apoptosis, which is responsible for the arrest of preimplantation embryo development [72]. Melatonin in maturation media improves the MMP and increases ATP content in bovine oocytes [20, 32], inhibits the opening of the mitochondrial permeability transition pore, and prevents the release of cytochrome c, thereby inhibiting caspase activation, decreasing apoptosis, and enhancing oocyte developmental competence [20, 73]. Similarly, a recent study has demonstrated melatonin's efficacy in enhancing developmental competence and optimizing mitochondrial function in buffalo oocytes [26]. However, the role of ROS impairment in cytoplasmic maturation of oocytes is well introduced; the distribution of ER in melatonin-treated oocytes is attributed to the ROS scavenging property, which has been introduced as the main characteristic of exogenous melatonin to prevent free-radical damage and cytotoxicity [74]. Melatonin reduces ROS formation and inhibits apoptosis [48, 73]. This is in addition to the increase in GSH content, which is important for the subsequent development of mature oocytes [75]; this is the reason for the improved quality of oocytes to develop into embryos. When OS happens in the cells, ROS instigates the externalization of phosphatidylserine, the early marker for apoptosis; melatonin reduces the OS by scavenging ROS to preserve the optimal function of mitochondria, homeostasis, and curtailing apoptosis [73]. Similarly, this mechanism has been well characterized in inferior bovine oocyte [31], mouse oocyte [76], and porcine oocyte [77], where melatonin improves oocyte quality by reducing OS during in vitro maturation.

The introduction of melatonin as an effective supplement of maturation media for the development of bovine oocytes is not reliable enough other than for evaluating the ability for fertilization and subsequent development of embryos. This was approved through the results of CR and BR, which were improved in the treated groups. In this context, melatonin exposure significantly enhanced the development of bovine embryos and increased the mean cell number of blastocysts produced after IVF [34] and the antiapoptotic properties of melatonin are suggested to be responsible [20]. We determined that the low concentrations of melatonin significantly increased the CR by 42% compared to nontreated oocytes, although high heterogeneity suggests that certain dosages in this category are more effective than others. The increase in CR and BR was observed in low-concentration groups but with high heterogeneity again. Moderate concentration increased the BR and HBR, the latter with no heterogeneity. The authors assume that high heterogeneity arises from the various numbers of oocytes that are subjected to different experiments, which is very common in ART studies in cattle.

However, the higher quality of oocytes affects positively the rates of cleavage and blastocyst formation by possessing greater developmental competence, which ensures the oocyte has the necessary cytoplasmic maturation and NM, metabolic activity, and molecular reserves to support early embryonic development [78]. Upon the results of this review, melatonin supplementation provides the bovine oocyte with the optimal intracellular environment and molecular machinery necessary for efficient cleavage and blastocyst formation, resulting in higher rates of embryo development. The transcription pattern of treated CCs, oocytes, and resultant blastocysts confirmed the mentioned mechanism; CYP11A1, CYP19A1, and StAR are involved in steroidogenesis [6, 79]; PTX3 and HAS1/2 regulate the expansion of CCs to predispose oocytes for proper maturation; receptors for LH (LHR) are crucial for the action of pituitary hormone to promote the meiotic maturation of oocytes; EGF regulates the action of LH; and the upregulation of these factors in treated CCs indicates the important role of melatonin in the progression of oocyte maturation process [34]. In oocytes, CAT, SOD1, and GPX help eliminate ROS directly [32]; growth differentiation factor 9 (GDF9) plays a key role in the development of CCs; MARF1 controls meiosis; DNMT1 maintains the normal methylation status, whereas TET promotes the demethylation of DNA; and melatonin treatment affects all these factors [32, 34]. The upregulation of antioxidant enzymes GPX1, SOD1, SOD2, and CAT is demonstrated in treated oocyte-derived blastocysts [20, 48]. Moreover, the expression of HSP70, a stress-response gene, increases to assist blastocysts in resisting apoptosis and OS [20, 80].

Overall, melatonin in IVM media improves nuclear and cytoplasmic maturation and affects the epigenetic modification to alleviate OS and early apoptosis, leading to subsequent promotion of the developmental quality and fertilization ability; these beneficial effects could be used to enhance the efficiency of in vitro–produced embryos in cattle.

The process of IVF has been considered to be manipulated for improved results. It is known that although OS significantly impacts reproductive processes, low levels of free radicals support gamete function and fertilization; however, excessive ROS can cause cellular damage, leading to apoptosis and developmental issues, negatively affecting IVF outcomes [81]. To mitigate this, antioxidant supplementation is proposed to reduce OS and improve embryo quality. In this context, the role of melatonin in enhancing sperm quality has been extensively investigated [82]; however, the sole study that investigated the effect of adding melatonin to IVF media on bovine embryo production [59] showed that 1 mM melatonin significantly reduced BRs. This negative effect may be due to the excessive reduction in ROS, which is necessary for normal fertilization processes [83]. Melatonin exhibits antiproliferative effects in various cell types, which can be both beneficial and detrimental depending on the context; high concentrations during IVF can have a deleterious effect by damaging embryo development, possibly due to excessive ROS production and depletion of reduced GSH [84]. This highlights the complex role of melatonin, which can vary significantly based on concentration and cellular environment. However, contrasting with findings in mice [85], lower concentrations (10–1000 nmol) did not improve bovine embryo production or quality. Although there are reports on incubating sperm with melatonin reducing their ability to bind to oocytes and decreasing polyspermy rates [64], suggesting that melatonin plays a significant role in regulating fertilization processes, melatonin supplementation at tested concentrations did not enhance in vitro bovine embryo production.

In the final section of this systematic review, the effect of melatonin-supplemented culture media on the progression of bovine presumptive zygotes was evaluated in different aspects of development. CR and BR data were subjected to meta-analysis to explore no significant effect of melatonin supplement on these critical events; however, HBR increased by 35% in the treatment group compared to the control group, meaning melatonin exposure is associated with a higher likelihood of hatching. Subgroup analysis based on melatonin concentration showed a positive effect of the moderate and low concentrations of melatonin on the BR and HBR. This is not surprising, given that high concentrations of melatonin have been shown to inhibit cell division, a critical process in development [86]; although the high concentrations cease division, the lower dosages improve the growth and development of blastocysts.

Another effect of melatonin translates into the quality of produced embryos in terms of the low apoptotic blastomeres. Although there are reports on the melatonin supplementation of culture media for the successful development of the ovine [87], porcine [21], and buffalo [24] preimplantation embryos, the results for the bovine embryos were not promising in this review. Melatonin's beneficial effects appear to be species-specific in this step of development. In cattle, the most prominent effect of melatonin during culture shows up in the quality of embryos and in reducing apoptosis. The quality of embryos is evaluated based on the number of apoptotic cells in blastocysts; apoptosis during preimplantation development is crucial for eliminating defective cells, but it can also lead to a significant loss of healthy cells, depending on the culture conditions [88]. In this regard, the mechanism of melatonin action is presented based on the expression of genes related to apoptosis and OS. OS is responsible for apoptosis, leading to lower blastocyst quality during culture, which is compensated by the downregulation of proapoptotic genes P53, BAX, and CASP3 and the upregulation of antiapoptotic BCL-2, XIAP, and MCL1 in treated blastocysts [20, 48, 61, 62]. Moreover, the downregulation of SHC1, which regulates the expression of BAX, is observed in the blastocysts derived from treated oocytes [48]. Alongside, melatonin upregulates antioxidant enzymes (GPX-4, CAT, and SOD1) and the antiapoptotic gene BCL-2; this reduces ROS production and apoptosis during embryo development, improving the quality of cultured embryos [61, 62]. Some developmentally important genes are influenced by melatonin in culture media as well; SLC2A1 is involved in the transport of glucose, DNMT1 is involved in epigenetic regulation by cytosine methylation of DNA, DNMT3A has a role in the methylation of most important loci, and OCC regulating the timing of blastocoel formation, CDH1 responsible for intercellular connectivity, and DSC2 essential for cell adhesion and blastocoel formation are all upregulated in the treated blastocysts, whereas the expression of AQP3, a transmembrane channel protein to regulate water flow, downregulated, which is believed to increase resistance to apoptosis [33, 61]. Collectively, melatonin supplement in culture media improves the quality of bovine embryos through modifications in multiple transcripts in blastocysts, which is reflected in the alleviation of OS and reduced apoptosis.

Preparing for this review, the in vivo studies were also considered to provide us with live birth rate outcomes of treated embryos; however, there were not enough results to be justified. Further research on live birth rates after transferring treated in vitro–produced embryos is needed to validate the commercial application of melatonin.

## 5. Conclusion

This review concludes that melatonin in maturation media is promising in the stage of application as it improves the quality of oocytes to give rise to more high-quality embryos able to progress better in development, whereas supplementation during the in vitro culture (IVC) stage seems to be too late to help embryo development, and the improvement in the quality of produced embryos is not superior to the higher proportion of embryo yield. However, more studies specifically on the in vivo transfer of embryos are needed to make a more concise conclusion for the commercial application of this supplement.

## Figures and Tables

**Table 1:** The characteristics of approved studies for analysis.

References	Melatonin dosage and supplementation stage	Groups and sample size	Included outcomes	Excluded outcomes
Takada et al, 2010 [40]	10^−9^ M IVM	ON1 = 209 ON2 = 171 One group	1. Nuclear maturation 2. Cleavage rate 3. Blastocyst rate 4. DNA damage in blastocysts	1. Data of no gonadotropin supplementation of media

Farahavar et al, 2010 [41]	10^−4^–10^−8^ M IVM	ON1 = 370 ON2 = 135 Three groups	1. Maturation rate 2. Cumulus expansion	

El-Raey et al, 2011 [7]	4 × 10^−7^–4 × 10^−8^ M IVM	ON1 = 360 ON2 = 114 Three groups	1. Maturation rate 2. Cumulus expansion 3. Mitochondrial activity and distribution pattern 4. Steroidogenesis of COCs 5. ROS levels in oocytes	1. Relative transcription of some genes not pertinent to this study 2-Data related to hydrogen peroxide

Sampaio et al, 2012 [42]	10^−6^–10^−13^ M IVM	ZN1 = 415 ZN2 = 75 Six groups	1. Cleavage rate 2. Blastocyst rate	1. Autoradiographic binding assay of the melatonin receptors and NQO_2_ enzyme

Takada et al, 2012 [43]	10^−9^ M IVM	ZN1 = 208 ZN2 = 209 One group	1. Cleavage rate 2. Blastocyst rate 3. Hatching/hatched blastocyst 4. DNA damage in cumulus cells	1. Data of media supplementation with melatonin without gonadotrophins

Cebrian-Serrano et al, 2013 [44]	10^−3^–10^−12^ M IVM	ZN1 = 1104 ZN2 = 602 Four groups	1. Cleavage rate 2. Blastocyst rate 3. Total cell number of blastocysts	1. Data of heat-stressed groups

Tian, 2014 [34]	10^−3^–10^−11^ M IVM	ZN1 = 1581 ZN2 = 461 Six groups	1. Oocyte maturation 2. Embryo development 3. Total cell number of blastocysts 4. Relative transcription in oocytes	1. Data of melatonin receptor detection in COCs by immunofluorescence and Western blotting 2. Melatonin in bovine follicular fluid 3. Data of luzindole treatment

Ahmed et al, 2016 [45]	10^−9^ M IVM	ON1 = 80 ON2 = 80 One group	1. Maturation rate 2. Cumulus expansion 3. COC diameter	1. Data of other supplements and heat-shock groups

Tsantarliotou et al, 2007 [46]	10^−3^–10^−5^ M IVM	ZN1 = 439 ZN2 = 155 Three groups	1. Cleavage rate 2. Blastocyst rate	

Rodrigues-Cunha et al, 2016 [47]	10^−6^, 10^−9^ M IVM	ON1 = 136 ON1 = 68 Two groups	1. Maturation rate 2. Relative transcription in oocytes and cumulus cells 3. Nuclear fragmentation in cumulus cells 4. ROS in oocytes 5. Cleavage rate 6. Blastocyst rate 7. Hatching rate	1. Data of maturation at 6, 12, and 18 h and negative control of no gonadotropin supplementation of media

Remiao et al, 2016 [48]	10^−6^–10^−12^ M, IVM	ON1 = 153 ON2 = 51 Three groups	1. Viability 2. Maturation rate 3. Cleavage rate 4. Blastocyst rate 5. ROS and GSH in oocytes 6. Total cell number and apoptosis in blastocysts 7. Relative transcript in blastocysts	1. The data of melatonin-loaded lipid-core nanocapsules

Marques et al, 2018 [19]	10^−7^–10^−11^ M, IVM and IVC	ZN1 = 975 ZN2 = 470 Four groups (IVM) ZN1 = 895 ZN2 = 437 Four groups (IVC)	1. GSH, ROS, and MMP in oocytes 2. Cleavage rate 3. Blastocyst rate and quality 4. Total cell number and apoptotic cells of blastocysts 5. GSH and ROS in blastocysts 6. Relative transcription in blastocysts	1. Data on supplementation throughout the IVP process

Wang et al, 2017 [49]	10^−11^–3 × 10^−11^ M IVM	ON1 = 798 ON2 = 251 Three groups	1. Maturation rate of oocytes 2. Steroid secretion of COCs in spent medium 3. Relative transcription in COCs 4. ROS level in embryos 5. Development of androgenetic embryos (cleavage, morula, and blastocyst rates)	1. Development of bovine parthenogenetic embryos (cleavage, morula, and blastocyst rates) 2. Melatonin receptors in embryos

Zhao et al, 2018 [32]	10^−7^–10^−11^ M IVM	ON1 = 10,069 ON2 = 3852 Three groups	1. Cytoplasmic maturation of MII oocytes (redistribution of mitochondria, cortical granules, and ER) 2. Intracellular glutathione (GSH) and ATP levels 3. Relative transcription in oocytes 4. Development results	1. Western blot data 2. Receptors 3. Luzindole group 4. IP3R1 distribution and expression of CD9 and Juno

Pang et al, 2018 [20]	10^−9^ M IVM	ON1 = 513 ON2 = 178 Three groups	1. Nuclear and cytoplasmic maturation 2. Blastocyst rate and apoptosis in blastocysts 3. Relative transcription in embryos	

An et al, 2019 [50]	10^−5^–10^−11^ M IVM	ON1 = 965 ON2 = 276 Four groups	1. Nuclear maturation of oocytes 2. Oxidative stress and cytoplasmic events 3. Development of embryos	1. Epigenetic modifications in embryos 2. RNA sequencing data 3. Data of SCNT

Cavallari et al, 2019 [51]	10^−6^ M IVM	ZN1 = 415 ZN2 = 415 One group	1. Cleavage rate 2. Blastocyst rate 3. ROS level in oocytes	1. Data of heat-shock and pro-oxidant treatment groups

El-Sheikh et al, 2019 [52]	10^−7^–10^−9^ M IVM	ZN1 = 600 ZN2 = 200 Three groups	1. Cleavage rate 2. Blastocyst rate	1. Data of SH6-treated oocytes

Lira et al, 2020 [53]	10^−7^–10^−11^ M IVM	ON1 = 340 ON2 = 116 Three groups	1. Maturation rate 2. Cleavage rate 3. Morula rate 4. Blastocyst rate	

Gutierres-Anez et al, 2021 [54]	10^−11^ M IVM	ZN1 = 209 ZN2 = 201 One group	1. Cleavage rate 2. Blastocyst rate 3. Total embryonic cells (ICM and TE)	1. The data of season, donor category, and the interaction of donor category by season effect on the cumulus–oocyte complexes yield in adult cows and prepubertal dairy cattle

Yaacobi-Artzi et al, 2020 [55]	10^−7^ M IVM	ON1 = 313 ON2 = 398 Two groups	1. Cumulus expansion 2. Maturation rate 3. Cleavage rate 4. Blastocyst rate	1. Data of heat-shocked groups

Su et al, 2021 [56]	10^−7^ -10^−10^ M IVM	ON1 = 1552 ON2 = 443 Five groups	1. Maturation rate 2. ROS levels in oocytes	1. Data of sperm 2. Data after fertilization

Cordova et al, 2022 [57]	2 × 10^−7^M IVM	NA	1. Relative transcription in cumulus cells and blastocysts	1. The development outcomes and quality of parthenogenetic blastocysts 2. Data of EGF supplement to medium 3. Immunofluorescence analysis

Tutt et al, 2023 [58]	10^−7^ M IVM	ZN1 = 105 ZN2 = 105 One group	1. The outcomes of embryo development	1. The results of media without CP 2. RNA-Seq analyses 3. Spent media analyses 4. DNA methylation, chromosomal errors, and aneuploidy in Day 8 blastocysts

Cheuqueman et al, 2015 [59]	10^−6^–10^−11^ M IVF	NA	1. Cleavage rate 2. Blastocyst rate 3. Total cell count	1. Data of sperm analysis

Papis, 2007 [60]	10^−4^ M IVC (2 days)	ZN1 = 100 ZN2 = 101 One group	1. Blastocyst rate of zygotes 2. Blastocyst rate of four-cell embryos	1. Data of culture at atmospheric oxygen

Wang et al, 2014, a [61]	10^−3^–10^−11^ M IVC	ZN1 = 1481 ZN2 = 681 Seven groups BN1 = 315 BN2 = 63 Five groups	1. Cleavage rate 2. Blastocyst rate 3. Hatched blastocysts 4. Total cell number of blastocysts 5. Relative transcription in blastocysts	1. The data of melatonin receptors 2. The data of luzindole-treated groups

Wang et al, 2014, b [33]	10^−7^ M IVC	ZN1 = 118 ZN2 = 117 One group	1. Cleavage rate 2. 8-cell stage rate 3. Blastocyst rate	1. Hatched-blastocyst rate of vitrified embryos 2. Relative transcription in vitrified blastocysts

Komninou, 2016 [62]	10^−6^–10^−12^ M IVC	ZN1 = 453 ZN2 = 165 Three groups	1. Cleavage rate 2. Blastocyst rate 3. Hatched blastocysts 4. Total cell number of blastocysts 5. ROS level and apoptosis in blastocysts 6. Relative transcription in blastocysts	1. Data of nanocapsule-coated groups

Hitit et al, 2023 [63]	10^−3^–10^−9^ M IVC	BN1 = 1846 BN2 = 709 Five groups	1. Blastocyst rat at D8 and D9 2. Hatched blastocyst at D9 3. Blastocyst cell number 4. Caspase-3/7 activity 5. Fragmentation of blastomeres	1. Data of heat-stressed embryos and groups of atmospheric oxygen tension

*Note:* ON1, number of oocytes in treatment; ON2, number of oocytes in control; ZN1, number of presumptive zygotes in treatment; ZN2, number of presumptive zygotes in control; BN1, number of blastocysts in treatment; BN2, number of blastocysts in control; GSH, glutathione; TE, trophectoderm.

Abbreviations: CPs, complex proteins; EGF, epidermal growth factor; ER, endoplasmic reticulum; ICM, inner cell mass; IVP, in vitro production; MMP, mitochondrial membrane potential; NA, not applicable; ROS, reactive oxygen species; SCNT, somatic cell nuclear transfer.

**Table 2:** Subgroup analysis of melatonin concentration in maturation media.

Outcome	Variable (melatonin concentration)	Number of studies	OR (95% CI)	*p* value	*I* ^2^%	*Q* statistics (*p*)
NM	Low	18	1.52 (1.16,1.99)	0.002	90.8	0.00
	Moderate	17	1.19 (0.95,1.50)	0.139	82.2	0.00
	High	1	0.88 (0.57,1.37)	0.578	—	—

CR	Low	30	1.42 (1.20, 1.69)	0.00	68.3	0.00
	Moderate	19	1.14 (0.95, 1.37)	0.155	61.2	0.00
	High	4	0.91 (0.60, 1.38)	0.645	76.6	0.005

BR	Low	30	1.35 (1.18, 1.55)	0.00	54.9	0.00
	Moderate	19	1.2 (1.03, 1.40)	0.019	53.8	0.003
	High	4	1.49 (0.61, 3.63)	0.381	87.2	0.00

HBR	Low	2	1.20 (0.55, 2.59)	0.649	65.8	0.054
	Moderate	2	1.69 (1.01, 2.83)	0.045	0.0	0.595

Abbreviations: BR, blastocyst rate; CR, cleavage rate; HBR, hatched-blastocyst rate; *I*^2^, heterogeneity; NM, nuclear maturation; OR, odds ratio.

**Table 3:** Descriptive synthesis of outcomes resulted from melatonin supplementation of maturation media.

Outcomes	Results in		
	CCs	Oocytes	Embryo development
Diameter of COCs	No change [45]		

Cumulus expansion	Fully expanded [7] Not expanded [7, 41, 45, 55] Reduced expansion [55]		

Live cells (%)	No change [48]		

DNA damage	No change [47] Reduced [43, 47]		No significant change was observed [19, 40]

Spindle disorganization		Reduced significantly [50]	

CG distribution		Higher peripheral and cortical distribution [20, 32]	

ER distribution		Higher normal distribution [32] No change [32]	

Mitochondria distribution		Higher normal distribution [7, 32] No change [7, 32]	

Mitochondrial activity		No change [7]	

MMP		Higher amount [20] No change [19] Lower amount of MMP [19]	

ATP level		Higher level [32]	

ROS level		No change [19, 47, 51] Lower level [7, 20, 32, 47, 48, 50, 56]	No change [19] Lower level [49]

GSH level		Higher level [20, 32] No change [19, 48]	No change [19]

Apoptosis		No change [20] Reduced [20, 50]	Reduced [20, 48, 50]

Two pronucleus			Increased [32]

Polyspermy			Decreased [32]

Unfertilized oocytes			Decreased [32]

Morula			No change [53]

Grade 1 blastocysts			No change [19] Decreased [19]

Grade 2 blastocysts			Increased [19] No change [19]

TCN			Increased [32, 34, 48, 50, 54] No change [19, 20, 34, 44]

ICM/TE			Increased [54] No change [50]

Antioxidant-related transcripts	Upregulation of CuZnSOD, MnSOD No change in GPX4, GPX1 Downregulation of CuZnSOD [47, 57]	Upregulation of CAT, SOD1, and GPX No change in CuZnSOD, MnSOD, and GPX4 [32, 47]	Upregulation of GPX, SOD1, SOD2, CAT, XIAP, MCL1 [19, 20, 48]

Apoptosis-related transcript			Upregulation of BCL-2 Downregulation of CASP3, BAX, and SHC1 [20, 48, 57]

Development-related transcripts		Upregulation of Tet1, Tet2, Tet3, GDF9, MARF1, DNMT1a Downregulation of Dnmt1 [32, 34]	Upregulation of HSP70 No change in OCT4, NANOG, PU5F1, SLC1A, SLC2A, SLC3A, HSPB1, and KRT8 [19, 20, 48, 57]

Steroidogenic-related transcripts	Upregulation of CYP11A1, CYP19A1, StAR [49]		

CC-specific transcripts	Upregulation of PTX3, HAS1, HAS2, LHR1, LHR2, EGFR No change in TNFAIP6, GREM1, and PTX3 [34, 57]		

Note: GSH, glutathione; TE, trophectoderm.

Abbreviations: CCs, cumulus cells; COCs, cumulus–oocyte complexes; ER, endoplasmic reticulum; ICM, inner cell mass; MMP, mitochondrial membrane potential; ROS, reactive oxygen species; TCN, total cell number.

**Table 4:** Subgroup analysis of melatonin concentration in culture media.

Outcome	Variable (melatonin concentration)	Number of studies	OR (95% CI)	*p* value	*I* ^2^%	*Q* statistics (*p*)
CR	Low	8	0.96 (0.78, 1.20)	0.738	36.0	0.142
	Moderate	6	1.172 (0.88, 1.56)	0.279	48.2	0.0607
	High	2	0.96 (0.67, 1.38)	0.834	0.0	0.496

BR	Low	11	1.26 (1.07, 1.49)	0.007	36.3	0.109
	Moderate	10	1.44 (1.15, 1.79)	0.001	54.4	0.020
	High	4	0.41 (0.17, 1.01)	0.052	85.4	0.00

HBR	Low	8	1.54 (1.12, 2.14)	0.009	27.8	0.206
	Moderate	6	1.57 (1.17, 2.11)	0.003	0.00	0.715
	High	4	0.54 (0.22, 1.32)	0.178	65.3	0.034

Abbreviations: BR, blastocyst rate; CR, cleavage rate; HBR, hatched-blastocyst rate; *I*^2^, heterogeneity; OR, odds ratio.

**Table 5:** Descriptive outcomes of melatonin effect in culture media.

Outcomes	Results
Four-cell embryos	No change [60]

Eight-cell embryos	Increased [33]; no change [61]

Grade 1 blastocysts	Increased [19]; no change [19]

Grade 2 blastocysts	Increased [19]; no change [19]

TCN	Increased [61, 62]; no change [19, 60, 61, 63]

Apoptosis	No change [19]; decreased [19, 62, 63]

Caspase activity	No change [63]

ROS level	No change [19]

GSH level	No change [19]

Antioxidant-related transcripts	Upregulation of GDF9, SOD1, SOD2, and CAT No change in SOD2, GPX, and PRDX5 [19, 61, 62]

Apoptosis-related transcripts	Upregulation of BCL-2 and MCL1 No change in SHC1, MCL1, P53, BAX, BAX/BCL-2 ratio Downregulation of BAX, caspase-3, SHC1 [61, 62]

Development-related transcripts	Upregulation of DNMT1A, SLC2A1, DSC2, DNMT3A, and HSP70 No change in OCT4, SOX2, NANOG, POU5F1, IFNT2, SLC2A1, SLC2A3, HSPB1, KRT8 [19, 61, 62]

*Note:* GSH, glutathione.

Abbreviations: ROS, reactive oxygen species; TCN, total cell number.

## Data Availability

All data are provided within the article and its supplementary data. Further inquiries can be directed to the corresponding author.

## Supporting Information

Supporting Information 1 Supporting 1: The details of the search strategy in three major scientific databases.

Supporting Information 2 Supporting 2: Publication bias for studies on melatonin supplementation during IVM, evaluated using Begg's and Mazumdar's adjusted rank correlation test and Egger's regression asymmetry test.

Supporting Information 3 Supporting 3: Publication bias for studies on melatonin supplementation during IVC, evaluated using Begg's and Mazumdar's adjusted rank correlation test and Egger's regression asymmetry test.

Supporting Information 4 Supporting 4: Research data file.

Supporting Information 5 Supporting 5: Abbreviation table.
