# Supplementary material for: Effect of Melatonin Supplementation on In Vitro Developmental Competence of Bovine Oocyte: A Systematic Review and Meta-Analysis
Source: Vet Med Int. 2025 Oct 24;2025:5351950. doi: 10.1155/vmi/5351950 (PMC12578564; doi:10.1155/vmi/5351950)
Supplement: Supporting Information 1 — Supporting 1: The details of the search strategy in three major scientific databases. [file 5351950.f1.docx]

**Search strategy**

**3, December, 2024**

**PubMed Keywords:**

**1- (cattle) OR (cow)) OR (bovine)) OR (zebu)) OR (yak)) OR (bos indicus)) OR (taurus)) OR (Holstein)) OR (dairy)) OR (beef)** [585,357](https://pubmed.ncbi.nlm.nih.gov/?term=%28%28%28%28%28%28%28%28%28cattle%29+OR+%28cow%29%29+OR+%28bovine%29%29+OR+%28zebu%29%29+OR+%28yak%29%29+OR+%28bos+indicus%29%29+OR+%28taurus%29%29+OR+%28Holstein%29%29+OR+%28dairy%29%29+OR+%28beef%29&sort=) results
2- (melatonin) AND (in vitro)) AND (oocyte): 282 results
3- (embryo) OR (development)) OR (maturation)) OR (meiosis)) OR (blastocysts)) OR (preimplantation)) OR (M phase)) OR (embryo) [7,947,287](https://pubmed.ncbi.nlm.nih.gov/?term=%28%28%28%28%28%28%28embryo%29+OR+%28development%29%29+OR+%28maturation%29%29+OR+%28meiosis%29%29+OR+%28blastocysts%29%29+OR+%28preimplantation%29%29+OR+%28M+phase%29%29+OR+%28embryo%29&sort=) results

**Combined:** 44 results

**Scopus**

## 1 - cattle OR bovine OR cow OR yak OR zebu OR Holstein: 719,757 documents found

## 2- melatonin AND in vitro AND oocyte: 380 documents found

## 3- maturation OR development OR meiosis OR blastocyst OR embryo: 9,925,548 documents found

**Combined queries**

## 1 AND 2 AND 3: 58 documents found

**WOS**

640,605 results from Web of Science Core Collection for:

1-(((((ALL=(cattle)) OR ALL=(bovine)) OR ALL=(cow)) OR ALL=(yak)) OR ALL=(zebu)) OR ALL=(holstein)

501 results from Web of Science Core Collection for:

2-((ALL=(melatonin)) AND ALL=(in vitro)) AND ALL=(oocyte)

10,681,352 results from Web of Science Core Collection for:

3-((((ALL=(maturation)) OR ALL=(development)) OR ALL=(meiosis)) OR ALL=(blastocyst)) OR ALL=(embryo)

**Combined Queries:**

#1 AND #2 AND #3: 123 results from Web of Science Core Collection
