# Supplementary material for: Effect of Melatonin Supplementation on In Vitro Developmental Competence of Bovine Oocyte: A Systematic Review and Meta-Analysis
Source: Vet Med Int. 2025 Oct 24;2025:5351950. doi: 10.1155/vmi/5351950 (PMC12578564; doi:10.1155/vmi/5351950)
Supplement: Supporting Information 3 — Supporting 3: Publication bias for studies on melatonin supplementation during IVC, evaluated using Begg's and Mazumdar's adjusted rank correlation test and Egger's regression asymmetry test. [file 5351950.f3.docx]

**Supplementary 3:**

Fig 1. The results of the Begg and Mazumdar adjusted rank correlation test (P =0.787), and Egger ҆s regression asymmetry test (P =0.847) for cleavage rate.

Fig 2. The results of the Begg and Mazumdar adjusted rank correlation test (P = 0.036), and Egger ҆s regression asymmetry test (P =0.162) for Blastocyst rate.

**Supplementary 3:**

Fig 3. The results of the Begg and Mazumdar adjusted rank correlation test (P =0.472), and Egger ҆s regression asymmetry test (P =0.990) for Hatched-blastocysts rate.
