# Supplementary material for: Effect of Melatonin Supplementation on In Vitro Developmental Competence of Bovine Oocyte: A Systematic Review and Meta-Analysis
Source: Vet Med Int. 2025 Oct 24;2025:5351950. doi: 10.1155/vmi/5351950 (PMC12578564; doi:10.1155/vmi/5351950)
Supplement: Supporting Information 4 — Supporting 4: Research data file. [file 5351950.f4.docx]

**1-Effects of Two Types of Melatonin-Loaded Nanocapsules with Distinct Supramolecular Structures: Polymeric (NC) and Lipid-Core Nanocapsules (LNC) on Bovine Embryo Culture Model**

# Komninou, 2016

**IVC**

|  | Treatment  **IVC** | No of  oocytes cultured | No of  zygotes cultured | Cleavage rate  (%) | **Blastocyst rate** | **Hatched blastocyst/blastocysts in each group (%) D9** |
| --- | --- | --- | --- | --- | --- | --- |
|  | Control | 192 | 165 | 141 (73%) | 63 (33%) | 8/27 (29.6)^a^ |
| IVC_A1 | MEL 10^-6^M | 175 | 158 | 128 (73) | 48 (27) | 16/29 (55)^b^ |
| IVC_A2 | MEL 10^-9^M | 176 | 154 | 124 (70) | 58 (33) | 17/28 (61)^b^ |
| IVC_A3 | MEL 10^-12^M | 158 | 141 | 117 (74) | 52 (33) | 18/30 (60)^b^ |

| Treatment  **IVC** | Number of  blastocysts  D7 | Total number of nuclei  (mean ± SEM) | Number of  TUNEL-stained nuclei  (mean ± SEM) | Apoptotic cell rate (%)  (mean ± SEM) |
| --- | --- | --- | --- | --- |
| Control | 16 | 95.6 ± 6.7^a^ | 10.7 ± 0.9^a^ | 10.1 ± 1,1^a^ |
| MEL10^-9^M | 13 | 150.2 ± 20,3^b^ | 8.5 ± 0.4^b^ | 5.6 ± 0.9^b^ |

(The level of **ROS** decreased in 4–8 cell stage embryos with treatment (10-9).

The total cell number of embryos increased with treatment and the apoptotic cell rate in blastocysts decreased with treatment.)

| **ROS** in treated 4-8 cell embryos | Significantly decreased↓ |
| --- | --- |
| Transcripts of **apoptosis-related genes** in blastocysts | BAX↓, CASP3↓, SHC1, MCL1 |
| Transcript levels for the **oxidative stress-related genes** in blastocysts | CAT↑, SOD2, GPX, PRDX5 |
| The relative abundance of mRNA for the **pluripotency-related genes** in blastocysts | OCT4, SOX2, NANOG |

**2- The effect of melatonin on bovine in vitro embryo development**

# Tsantarliotou, 2007

supplementation of the IVM medium

| ID | Groups | COCs N | Cleaved N (%) | Blastocysts N (%) |
| --- | --- | --- | --- | --- |
|  | Control | 155 | 110 (71.0) | 64 (41.3) |
| IVM_A1 | 10 µM Melatonin | 151 | 110 (72.8) | 51 (33.8) |
| IVM_A2 | 100 µM Melatonin | 160 | 116 (72.5) | 63 (39.4) |
| IVM_A3 | 1 µM Melatonin | 128 | 93 (72.7) | 49 (38.3) |

No difference

**3- Melatonin in maturation media fails to improve oocyte maturation, embryo development rates and DNA damage of bovine embryos**

# Takada, 2010

**IVM**

|  | Culture condition (IVM) | No. of oocytes* | Nuclear maturation rates % (n) NS |
| --- | --- | --- | --- |
|  | (control) | 171 | 88.9 (152) |
| IVM_B1 | 10^–9^ M | 209 | 89.0 (198) |

|  | Culture condition | N of oocytes | Cleavage rate% (n) NS | Blastocyst rate% (n) NS |
| --- | --- | --- | --- | --- |
|  | (control) | 209 | 85.7 (178) | 43.5 (91) |
| IVM_B1 | 10–9 M MEL | 208 | 85.7 (178) | 42.8 (89) |

The extent of **DNA damage** in cattle **blastocysts** determined by comet assay was **not** different between groups.

**4- Effect of melatonin on in vitro maturation of bovine oocytes**

**Abbas Farahavar, 2010**

IVM

|  | Melatonin concentrations (µM | Number of COCs | Nuclear maturation, n(%)  (Mean ± S.E) |
| --- | --- | --- | --- |
|  | 0 | 135 | 98 (72.24 ± 1.17) ab |
| IVM_C1 | 0.01 µM | 127 | 93 (73.11 ± 1.17) a |
| IVM_C2 | 1 µM | 116 | 83 (70.68 ± 1.17) b |
| IVM_C3 | 10 µM | 127 | 83 (65.24 ± 1.17) c |

Data of cumulus expansion was not different between groups.

**5- Beneficial effects of melatonin on in vitro bovine embryonic development are mediated by melatonin receptor 1**

**Wang, 2014**

IVC, (mean ± S.E.M)

|  | Melatonin  IVC (early 2 days) | N of embryos | Cleavage rate(n)  NS | Blastocyst rate | Hatched blastocyst rate | Cell number/blastocyst |
| --- | --- | --- | --- | --- | --- | --- |
|  | 0 | 200 | (150) 74.88±3.31 | (60) 29.9 ± 3.3% a | (26) 13.0 ± 3.1% a | 92.05±1.97 |
| IVC_B1Early | 10^-3^ | 200 | (145) 72.51±3.79 | (49) 24.28±0.95 | (27) 13.33±0.21 | 86.24±2.63 |
| IVC_B2Early | 10^-5^ | 200 | (159) 79.62±3.79 | (67) 33.33±2.86 | (30) 15.23±2.38 | 89.63±1.98 |
| IVC_B3Early | 10^-7^ | 200 | (158) 79.14±3.32 | (79) 39.7 ± 3.7% b | (44) 22.1 ± 2.5% b | 95.01±1.97 |
| IVC_B4Early | 10^-9^ | 200 | (164) 81.99±3.31 | (71) 35.71±3.33 | (34) 17.14±3.81 | 95.77±1.98 |
| IVC_B5Early | 10^-11^ | 200 | (148) 73.93±4.26 | (63) 31.42±1.91 | (25) 12.38±0.95 | 91.28±2.63 |

|  | Melatonin  IVC (late 6 days) | N of embryos | Blastocyst rate | Hatched blastocyst rate | Cell number/blastocyst |
| --- | --- | --- | --- | --- | --- |
|  | 0 | 63 | (13) 20.1 ± 2.3% a | (4) 6.7 ± 0.8% a | 81.82±3.75 |
| IVC_B1Late | 10^-3^ | 63 | (11) 18.16±2.24 | (3) 4.7±1.8 | 77.55±5.02 |
| IVC_B2Late | 10^-5^ | 63 | (19) 29.82±4.03 | (6) 10.31±2.02 | 80.15±3.75 |
| IVC_B3late | 10^-7^ | 63 | (21) 32.73±2.92 | (7) 10.98±1.8 | 84.63±3.13 |
| IVC_B4Late | 10^-9^ | 63 | (22) 35.6 ± 6.3% b | (10) 15.9 ± 2.2% b | 85.36±4.38 |
| IVC_B5Late | 10^-11^ | 63 | (17) 26.68±6.72 | (5) 8.74±2.24 | 82.97±3.75 |

|  | Melatonin  IVC (combined)  Entire culture | N of embryos | Cleavage rate | Blastocyst rate | mean cell number/ blastocyst |
| --- | --- | --- | --- | --- | --- |
|  | 0 | 219 | (144) 65.75±2.26a | (42) 19.34±2.83a | 67.03±2.6a |
| IVC_B6 | 10^-7 and^ 10^-9^ | 219 | (169) 77.33±0.1b | (85) 38.83±0.1b | 97.23±1.3b |

|  | Melatonin  IVC | N of embryos | Cleavage rate  NS | 8-Cells embryos | Blastocyst rate | Hatched blastocyst rate | Cell number/blastocyst |
| --- | --- | --- | --- | --- | --- | --- | --- |
|  | 0 | 262 | (212) 80.88±1.56a | a | (61) 23.1 ± 2.4%b | (21) 8.0 ±1.3%b | 109.7 ± 4.7b |
| IVC_B7 | melatonin10^-7^ | 262 | (213) 81.06±2.07a | a | (82) 31.4 ± 1.7%a | (35) 13.4 ± 2.4%a | 133.2 ± 5.6a |

The results showed that only MT1 activation is involved in bovine embryo development.

| Relative transcription of **anti-oxidative enzymes** in blastocysts | GPX4 ↑, SOD1↑ , |
| --- | --- |
| Relative transcription of **apoptosis-related genes** in blastocysts | P53, BAX↓ , caspase-3↓, Bcl-2↑ |
| Relative transcription of **developmentally important** **genes** in blastocysts | DNMT1A↑, SLC2A1↑, SLC2A3, IFNT2, DSC2↑ |

SLC2A1 (glucose transporter 1)

DNMT1A (DNA methyltransferase 1a)

**6- Supplementation of IVF medium with melatonin: effect on sperm functionality and in vitro produced bovine embryos**

**Cheuqueman, 2014**

**IVF**

Gametes were coincubated in an IVF medium supplemented with 1, 0.01, 0.1, and 1 mmol melatonin as well as 2, 10 100, and 1000 nmol melatonin.

No significant difference was observed in cleavage, blastocyst rates, and total embryo cell count. However, a significant decrease in blastocyst rate was observed at 1 mmol melatonin.

**7- Comparative efficacy of antioxidant retinol, melatonin, and zinc during in vitro maturation of bovine oocytes under induced heat stress**

**AHMED, 2016**

IVM

|  | Melatonin concentration IVM | Number of COCs | Metaphase 2 % (n) |
| --- | --- | --- | --- |
|  | 0 | 80 | 67.14 (54) |
| IVM_D1 | 1 nM | 80 | 66.96 (54) |

**Cumulus expansion** was not different between treatment and control groups.

The diameter of grade A COCs increased significantly in treatment compared to the ctrl group.

**8- Use of Melatonin in the In Vitro Production of Bovine Embryos**

**Lira,2020**

IVM

|  | M concentration (µM) | Number of COCs | Maturation rate % |
| --- | --- | --- | --- |
|  | 0 | 116 | 49.14% (57/116)a |
| IVM_E1 | 10^-5^ | 126 | 29.27% (36/126)b |
| IVM_E2 | 10^-3^ | 113 | 45.13% (51/113)a |
| IVM_E3 | 10^-1^ | 101 | 17.82% (18/101)b |

|  | M concentration | Number of COCs | Cleavage rate | Morula rate | Blastocyst rate |
| --- | --- | --- | --- | --- | --- |
|  | 0 | 83 | 32 (38.6a%) | 30 (36.1ab%) | 15 (18.1b%) |
| IVM_E1 | 10^-5^ | 51 | 27 (52.9a%) | 27 (52.9a%) | 18 (35.3a%) |
| IVM_E2 | 10^-3^ | 88 | 36 (40.9a%) | 31 (35.2b%) | 8 (9.1b%) |
| IVM_E3 | 10^-1^ | 77 | 15 (19.5b%) | 15 (19.5c%) | 6 (7.8b%) |

|  | Morula | Initial blastocyst | Blastocyst | Expanded blastocyst | TOTAL |
| --- | --- | --- | --- | --- | --- |
| Control | 15 (50.0%)ab | 7 (23.3%)ab | 6 (20.0%)a | 2 (6.7%)a | 30 |
| 10^–5^ μM | 9 (33.3%)b | 12 (44.4%)a | 4 (14.8%)a | 2 (7.4%)a | 27 |
| 10^–3^ μM | 23 (74.2%)a | 3 (9.7%)b | 4 (12.9%)a | 1 (3.2%)a | 31 |
| 10^–1^ μM | 9 (60.0%)ab | 4 (26.7%)ab | 1 (6.7%)a | 1 (6.7%)a | 15 |

**???Table 4: The development of grade 1 COCs was not influenced by the melatonin treatment in IVM media but ??? not included in SPSS**

**9-** Melatonin Protects Bovine Embryos from Heat Stress and Oxygen Tension and Improves Embryo Production In vitro

**Hitit, 2023**

**IVC- two experiments**

|  | Groups  Melatonin in IVC | Zygotes (n) | Blastocyst rate % D8 (n) | Blastocyst rate % D9 | Hatched Blastocyst Day 9 (%) |
| --- | --- | --- | --- | --- | --- |
|  | 0 | 394 | (59) 14.9 b | 17.1 b | (19) 4.7 b |
| IVC_C1 | 10^-3^ | 408 | (7) 1.6 c | 2.3 c | (0) 0.0 c |
| IVC_C2 | 10^-6^ | 400 | (86) 21.4 a | 24.9 a | (26) 6.6 a |
| IVC_C3 | 10^-9^ | 414 | (90) 21.8 a | 23.3 a | (21) 5.1 a |

Melatonin treatment didn’t change the **Caspase3/7 activity** in Eight-cell embryos (30, each group) compared to the control group.

|  | Groups  Melatonin in IVC | Zygotes (n) | Blastocyst rate (n) % D8 | Blastocyst cell number (NS) | DNA fragmentation (TUNLE+Blastomers) |
| --- | --- | --- | --- | --- | --- |
|  | 0 | 312 | (48) 15.32±1.33a | 107.02±8.4 | 8.43±1.35a |
| IVC_C4 | 10^-6^ | 312 | (58) 18.61±1.09a | 107.1±6.16 | 4.83±0.56b |
| IVC_C5 | 10^-9^ | 312 | (71) 22.86±2.89b | 118.38±7.84 | 2.92±0.34b |

the Caspase3/7 activity in Eight-cell embryos (30 each group) was not different.

**Apoptosis** decreased in treated embryos.

**10-** **Melatonin Improves the Quality of In Vitro Produced (IVP) Bovine Embryos: Implications for Blastocyst Development, Cryotolerance, and Modifications of Relevant Gene Expression**

**Wang, 2014b**

**IVC, (mean ± SEM)**

|  | **Melatonin in IVC** | **N of zygotes** | **Cleavage rate (n)** | **8-cell rate** | **Blastocyst rate at D7** |
| --- | --- | --- | --- | --- | --- |
|  | **0** | **117** | **(97) 82.50±1.12%a** | **(94) 80.00±1.29%a** | **(31) 26.67±1.05%a** |
| **IVC_D1** | **10-7 M** | **118** | **(104) 87.78±1.02%b** | **(100) 84.45±0.93%b** | **(45) 38.33±2.21%b** |

| **Quality assessment after freeze-thaw** | **12h** | **24h** | **36h** | **48h** | **60h** | **72h** |
| --- | --- | --- | --- | --- | --- | --- |
| **Hatched blastocyst rate** | **-** | **↑** | **↑** | **↑** | **↑** | **↑** |
| **Mortality rate** | **-** | **-** | **-** | **↓** | **↓** | **↓** |

| **Relative transcription of developmentally important genes in vitrified blastocysts** | **DNMT3A↑, SLC2A3, IFNT2, OCC↑, TJP1, HSPA1A, AQP3↓, CDH1↑** |
| --- | --- |

**11- Melatonin effect on bovine embryo development in vitro in relation to oxygen concentration**

**Papis, 2007**

**IVC**

|  | Treatment  IVC | No.of zygotes cultured | Cleavage rate | Ratio (n) of ‡4-cell embryos | Blastocyst rate | Hatching/hatched blastocyst rate |
| --- | --- | --- | --- | --- | --- | --- |
|  | 0 | 101 | 72 | 64.4 (65) | 44/65 (67.7%)a | 36/65 (55.4%) |
| IVC_E1 | 10^−4^ M  Melatonin | 100 | 74 | 65.0 (65) | 31/65 (47.7%)b | 24/65 (36.9%) |

| Treatment  IVC | No. of embryos examined | Cell Number  mean ± SE |
| --- | --- | --- |
| 0 | 9 | 166.0 ± 17.0 |
| 10^−4^ M | 6 | 172.0 ± 13.9 |

**12- Evidence of Melatonin Synthesis in the Cumulus Oocyte Complexes and its Role in Enhancing Oocyte Maturation In Vitro in Cattle**

**EL-RAEY,2011**

IVM

|  | Melatonin ng/ml  (M) | Melatonin (ng/ml) | No. of oocytes examined | MII (% ± SEM) |
| --- | --- | --- | --- | --- |
|  | 0 | 0 | 114 | 56 (49.1 ± 3.8)b |
| IVM_F1 | 10 (4.3*10^-8^M) | 10 | 127 | 93 (73.2 ± 2.2)a |
| IVM_F2 | 50 (2.15*10^-7^M) | 50 | 110 | 74 (67.2 ± 4.5)a |
| IVM_F3 | 100(4.3*10^-7^M) | 100 | 123 | 63 (51.2 ± 3.7)b |

Melatonin supplementation significantly increased the proportion of fully **expanded** cumulus cells.

The level of **progesterone** and **estrogen** in spent media was not significantly different.

**Mitochondrial activity** was not different but the **distribution of active mitochondria** increased in treated oocytes.

The level of **ROS** significantly decreased in treated oocytes.

| Melatonin ng/ml  (M) | No. of oocytes examined | Mitochondrial distribution type (% mean SEM)  Peripheral | Mitochondrial distribution type (% mean SEM)  Dispersed |
| --- | --- | --- | --- |
| 0 | 54 | 57.5 2.4a | 42.4 2.4b |
| 10 (4.3*10^-5^M) | 60 | 33.3 6.5b | 66.6 6.5a |
| 50 (2.15*10^-4^M) | 55 | 28.0 4.8b | 71.9 4.8a |
| 100(4.3*10^-4^M) | 55 | 50.9 3.3a | 49.0 3.3b |

| Melatonin ng/ml  (M) | Melatonin (ng/ml) | Number of oocytes | (ROS) Relative total fluorescence values in oocytes after staining* |
| --- | --- | --- | --- |
| 0 | 0 | 53 | 100.00 0.00a |
| 10 (4.3*10^-5^M) | 10 | 52 | 63.2 5.2b |
| 50 (2.15*10^-4^M) | 50 | 53 | 61.6 5.3b |
| 100(4.3*10^-4^M) | 100 | 52 | 62.3 5.1b |

**13- MT3 melatonin binding site, MT1 and MT2 melatonin receptors are present in oocyte, but only MT1 is present in bovine blastocyst produced in vitro**

**Rafael V Sampaio, 2012**

IVM, (mean ± standard error of the mean (SEM))

|  | Melatonin concentration | Number of oocytes | Cleavage at day2(n) | Blastocyst at day 7(n) |
| --- | --- | --- | --- | --- |
|  | 0 | 75 | (40) 52.9 ± 2.8 a | (23) 30.9 ± 3.9a |
| IVM_G1 | 0.1 pM | 60 | (34) 57.3 ± 6.6 a | (19) 32.0 ± 6.6b |
| IVM_G2 | 1 pM | 75 | (47) 62.1 ± 5.0 a | (33) 43.7 ± 2.3c |
| IVM_G3 | 0.01 nM | 75 | (40) 52.7 ± 1.8 a | (26) 34.2 ± 3.6a, b |
| IVM_G4 | 0.1 nM | 75 | (30) 39.4 ± 4.0 b b | (15) 19.8 ± 3.7a, |
| IVM_G5 | 1 nM | 55 | (22) 40.4 ± 8.7 b | (16) 30.0 ± 3.6b |
| IVM_G6 | 1 μM | 75 | (26) 34.4 ± 3.5 b | (19)25.5 ± 1.3d |

**14-Effect of melatonin on DNA damage of bovine cumulus cells during in vitro maturation (IVM) and on in vitro embryo development**

**L.** **Takada, 2012**

IVM

|  | Groups | Number of oocytes | Cleavage rate ±SEM % (n) | Blastocyst D7 | Blastocyst D8 | Hatching/hatched blastocyst D8 |
| --- | --- | --- | --- | --- | --- | --- |
|  | Ctrl | 209 | (179) 85.7 ± 2.4 | (47) 22.5 ± 3.6 | (71) 33.9 ± 2.6 | (45) 21.6 ± 3.0 |
| IVM_H1 | Treatment (10-9 M melatoninIVM) | 208 | (178) 85.7 ± 2.4 | (43) 20.8 ± 3.6 | (72) 35.0 ± 2.6 | (48) 23.1 ± 3.0 |

Not significant

The extent of **DNA damage in CCs** was significantly reduced by melatonin supplementation during in vitro maturation (Comet assay).

**15-Effects of melatonin on production of reactive oxygen species and developmental competence of bovine oocytes exposed to heat shock and oxidative stress during in vitro maturation Fernanda de** **Cavallari, 2019**

**IVM**

|  | Melatonin concentration | Number of COCs | ROS (pixel intensity)  N=41-72 | Cleavage rate (n) | Blastocyst rate D7.5 |
| --- | --- | --- | --- | --- | --- |
|  | 0 | 415 | 710.682a | (273) 65.81%a | (111) 26.65a |
| IVM_I1 | 10^-6^M | 415 | 560.673a | (280) 67.46%a | (123) 29.74a |

The level of **ROS** didn’t change significantly.

16-**Effects of melatonin during IVM in defined medium on oocyte meiosis, oxidative stress, and subsequent embryo development**

**Rodrigues-Cunha, 2016**

**IVM**

|  | Melatonin concentration | Number of oocytes | Nuclear maturation M2 % (n) |
| --- | --- | --- | --- |
|  | 0 | 68 | (47) 69.7±4.45 |
| IVM_J1 | 10^-6^ M | 68 | (45) 65.95±6.08 |
| IVM_J2 | 10^-9^ M | 68 | (35) 52.08±6.48 |

| Melatonin concentration | Number of oocytes | ROS, Fluorescence intensity (arbitrary units); mean ± SEM |
| --- | --- | --- |
| 0 | 64 | 45.78 ± 0.7a |
| 10^-6^ M | 72 | 43.52 ± 0.6b |
| 10-^9^ M | 65 | 45.18 ± 0.6ab |

| Melatonin concentration | Number of oocytes | Cleavage, n (%± SEM) | Blastocyst, n (%± SEM) | Hatched blastocysts, n (%± SEM) |
| --- | --- | --- | --- | --- |
| 0 | 165 | 144 (87.3 ± 2.6) | 89 (53.9 ± 3.9) | 85 (95.5 ± 2.2) |
| 10^-6^ M | 152 | 132 (86.8 ± 2.7) | 76 (50.6 ± 4.1) | 73 (96.1 ± 2.2) |
| 10^-9^ M | 152 | 127 (83.6 ± 3.0) | 74 (48.7 ± 4.1) | 67 (90.5 ± 3.4) |

Not significant

| Relative transcription of antioxidant enzymes in **cumulus** cells | Cu,ZnSOD↑, MnSOD↑, GPX4 |
| --- | --- |
| Relative transcription of antioxidant enzymes in **oocytes** | Cu,ZnSOD, MnSOD, GPX4 |

10^-6^ M melatonin in IVM media significantly reduced the **level of ROS in oocytes** and **nuclear fragmentation in cumulus cells** compared to control.

**17-Melatonin delivery by nanocapsules during in vitro bovine oocyte maturation decreased the reactive oxygen species of oocytes and embryos**

**Remiao, 2016**

**IVM**

|  | Melatonin concentration | Number of COCs | The percentage of live cells | Nuclear Maturation rate % mean±SEM (n) |
| --- | --- | --- | --- | --- |
|  | 0 | 51 | 100% | (32) 63.51% ± 4.59a |
| IVM_K1 | 10^-6^ | 51 | 94.9% | (47) 92.53% ± 4.58 b |
| IVM_K2 | 10^-9^ | 51 | 100% | (42) 81.64% ± 6.69 b |
| IVM_K3 | 10^-12^ | 51 | 100% | (44) 86.42% ± 9.43 b |

The percentage of live cells was not different between groups.

| Melatonin concentration  IVM | Number  of oocytes | Cleavage rate%  (N) mean±SEM | Blastocyst rate%  (n) mean±SEM |
| --- | --- | --- | --- |
| 0 | 74 | (53) 71.92% ± 3.75a | (26) 35.26% ± 2.42a |
| 10^-6^ | 74 | (64) 86.80% ± 1.95b | (31) 42.09% ± 2.33 b |
| 10^-9^ | 74 | (58) 77.92% ± 3.15 b | (29) 39.56% ± 2.16 b |
| 10^-12^ | 74 | (57) 77.29% ± 2.77 b | (32) 43.75%± 1.83b |

| Melatonin concentration IVM | **mean cell number/blastocyst** | **apoptotic cell number/blastocyst** | **Level of ROS in oocytes** | **GSH in oocytes** |
| --- | --- | --- | --- | --- |
| 0 | 66.84 ± 9.06a | 21.75 ± 3.16a | 59.05 ± 5.81a | 157.90 ± 6.63a |
| 10^-9^ | 102.27 ± 10.71b | 13.33 ± 2.13b | 43.00 ± 2.93b | 147.10 ± 7.10a |

Treatment significantly reduced the **apoptotic cells in blastocysts**, decreased **ROS** level, and no effect on **GSH** in oocytes.

| Transcription of **oxidative stress-related genes** in blastocysts | GPX↑, SOD1↑, SOD2↑, CAT↑ |
| --- | --- |
| Transcription of **apoptosis-related genes** in blastocysts | BAX↓, casp3↓, MCL1↑, SHC1↓ |
| Transcription of **pluripotency genes** in blastocysts | NANOG, POU5F1 , SOX2 |

18-**Melatonin reduces apoptotic cells, SOD2 and HSPB1 and improves the in vitro production and quality of bovine blastocysts**

**TC Marques, 2018**

IVM, IVC (Data are presented as mean ± standard deviation)

|  | Melatonin concentration  IVM | N of COCs | MMP  N=140 | ROS  N=152 | GSH  N=152 | Cleavage | Blastocyst | Quality  Grade 1 blast | Quality  Grade 2 blast |
| --- | --- | --- | --- | --- | --- | --- | --- | --- | --- |
|  | 0 | 254 | a | a | a | 75.6 (192/254)b | 39.8 (101/254)a | 40.6 (41/101)ab | 38.6 (39/101)b |
| IVM_L1 | 10^−7^ M | 260 | c | a | a | 74.6 (194/260)b | 36.2 (94/260)a | 27.7 (26/94)b | 36.2 (34/94)b |
| IVM_L2 | 10^−9^ M | 253 | a | a | a | 88.9 (225/253)a | 42.3 (107/253)a | 43.0 (46/107)a | 34.6 (37/107)b |
| IVM_L3 | 10^−11^ M | 261 | b | a | a | 83.1 (217/261)a | 44.4 (116/261)a | 10.3 (12/116)c | 69.0 (80/116)a |

The levels of **ROS** and **GSH** in oocytes were not different between groups. **MMP** in oocytes of 10^-7^ and 10^-11^ groups was significantly lower than the control.

|  | Treatment  IVC | Number of oocytes | Cleavage (%) | Blastocyst (%) | Blastocyst quality (%) Grade I | Blastocyst quality (%) Grade II |
| --- | --- | --- | --- | --- | --- | --- |
|  | Control | 221 | 79.6 (176/221)ab | 37.1 (82/221)bc | 39.9 (32/82)b | 34.1 (28/82)a |
| IVC_F1 | IVC+ 10^-7^ | 223 | 75.8 (169/223)bc | 43.5 (97/223)ab | 56.7 (55/97)ac | 28.9 (28/97)a |
| IVC_F2 | IVC+ 10^-9^ | 228 | 84.2 (192/228)a | 47.4 (108/228)a | 57.4 (62/108)ac | 27.8 (30/108)a |
| IVC_F3 | IVC+ 10^-11^ | 228 | 70.6 (161/228)c | 31.1 (71/228)c | 50.7 (36/71)bc | 35.2 (25/71)a |

The proportion of grade 1 blastocysts increased with melatonin treatment (10^-7^ and 10^-9^). Total cell number was not different but apoptosis was decreased in the 10^-9^ group.

| Treatment  IVC | Number of blastocysts | Total cell number | Number of apoptotic cells (%) |
| --- | --- | --- | --- |
| Control | 20 | 115.9 ± 4.0a | 6.1 ± 0.6 (5.3)b |
| IVC+ 10^-7^ | 20 | 115.1 ± 3.8a | 6.3 ± 0.5 (5.5)b |
| IVC+ 10^-9^ | 20 | 103.3 ± 4.1a | 3.8 ± 0.6 (3.6)a |
| IVC+ 10^-11^ | 20 | 111.4 ± 4.0a | 5.1 ± 0.6 (4.6)ab |

|  | Treatment | Number of oocytes | Cleavage (%) | Blastocyst (%) | Blastocyst quality (%) Grade I | Blastocyst quality (%) Grade II | ROS in blastocysts | GSH in blastocysts |
| --- | --- | --- | --- | --- | --- | --- | --- | --- |
|  | Control | 216 | 83.3 (180/216)a | 41.7 (90/216)b | 35.6 (32/90)b | 34.4 (31/90)a | a | ab |
| IVM_L4 | IVM + 10^−9^ | 201 | 86.6 (174/201)a | 47.3 (95/201)ab | 44.2 (42/95)b | 31.6 (30/95)a | a | b |
| IVC_F4 | IVC + 10^−9^ | 216 | 82.9 (179/216)a | 52.8 (114/216)a | 52.6 (60/114)a | 28.9 (33/114)a | a | a |
|  | IVM/IVC + 10^−9^ | 206 | 79.6 (164/206)a | 42.2 (87/206)b | 46.0 (40/87)b | 36.8 (32/87)a | b↑ | ab |

GSH in blastocysts was not different. ROS in blastocysts increased in the group with the entire melatonin treatment.

The total cell number was not different but the number of apoptotic cells was lower in IVC + 10^−9^ group

|  | Treatment | Number of blastocysts | Total cell number | Number of apoptotic cells (%) |
| --- | --- | --- | --- | --- |
|  | Control | 16 | 135.0 ± 7.2a | 6.5 ± 0.6 (4.8)b |
|  | IVM + 10^−9^ | 15 | 130.6 ± 6.7a | 5.3 ± 0.6 (4.0)b |
| IVC_F4 | IVC + 10^−9^ | 18 | 116.6 ± 5.0a | 3.0 ± 0.5 (2.5)a |
|  | IVM/IVC + 10^−9^ | 15 | 130.2 ± 7.3a | 5.4 ± 1.0 (4.1)b |

| Relative transcription in blastocysts (IVM treatment) | SLC2A1, SLC2A3, SOD2, HSPB1, KRT8 |
| --- | --- |
| Relative transcription in blastocysts (IVC treatment) | SLC2A1, SLC2A3, SOD2↑, HSPB1, KRT8 |
| Relative transcription in blastocysts (IVM+IVC treatment) | SLC2A1, SLC2A3, SOD2, HSPB1, KRT8 |

(genes related to metabolism (SLC2A1, SLC2A3), oxidative stress (HSPB1, SOD2) and placentation (KRT8))

**19- The effects of melatonin on bovine uniparental embryos development in vitro and the hormone secretion of COCs**

**Wang, 2017**

**IVM**

|  | Melatonin (pmol/ml) | COCs (n) | Maturation rate of oocytes (n) % ± s.e.m |
| --- | --- | --- | --- |
|  | 0 | 251 | (104) 41.60 ± 14.31a |
| IVM_N1 | 10 | 285 | (146) 51.32 ± 7.32b |
| IVM_N2 | 20 | 260 | (149) 57.47 ± 5.65b |
| IVM_N3 | 30 | 253 | (171) 67.40 ± 3.80c |

| Melatonin pmol/mL (M) | Parthenogenetic embryos (n) | Rate of cleavage (% ± s.e.m ) | Rate of morula (% ± s.e.m) | Rate of blastocyst (% ± s.e.m) |
| --- | --- | --- | --- | --- |
| 0 | 133 | 44.44 ± 6.77a (59/133) | 30.86 ± 6.63a (41/133) | 14.43 ± 7.48a (9/59) |
| 10 (10nM) | 140 | 67.25 ± 13.35b (95/140) | 48.21 ± 10.65b (68/140) | 19.55 ± 3.55a (19/95) |
| 20 (20nM) | 149 | 69.03 ± 9.85b (103/149) | 51.33 ± 20.22b (45/149) | 20.01 ± 9.57a (21/103) |
| 30 (30nM) | 145 | 66.76 ± 11.54b (96/145) | 52.96 ± 10.73b (46/145) | 22.02 ± 4.44b (21/96) |

| Melatonin pmol/mL (M) | Androgenetic embryos (n) | Rate of cleavage (% ± S.E.M ) | Rate of morula (% ± S.E.M) | Rate of blastocyst (% ± S.E.M) |
| --- | --- | --- | --- | --- |
| 0 | 104 | 55.46 ± 9.03a (58/104) | 19.22 ± 1.36a (20/104) | 0a (0/58) |
| 10 | 86 | 70.14 ± 4.34a (60/86) | 35.42 ± 5.51b (30/86) | 13.10 ± 4.44b (8/60) |
| 20 | 110 | 76.38 ± 3.90bc (84/110) | 38.26 ± 2.13b (42/110) | 14.26 ± 3.13b (12/84) |
| 30 | 112 | 77.25 ± 4.28c (86/112) | 37.58 ± 1.42b (42/112) | 14.06 ± 1.46b (12/86) |

The melatonin treatment decreased the level of **ROS** in treated **blastocysts**.

The concentration of **estradiol** and **progesterone** in spent media increased with melatonin treatment.

The results revealed the **presence of MT1 and MT2** in early bovine parthenogenetic and androgenetic embryos.

| Relative transcription of steroidogenic-related genes in COCs | CYP11A1↑, CYP19A1↑ , StAR↑ |
| --- | --- |

**20-Melatonin Abrogates the Anti-Developmental Effect of the AKT Inhibitor SH6 in Bovine Oocytes and Embryos**

**Marwa El Sheikh, 2019**

**IVM**

|  | Melatonin concentration | N of oocytes | Cleavage rate±SEM % at day 4 (n) | Blastocyst rate % at day 8 (n) |
| --- | --- | --- | --- | --- |
|  | 0 | 200 | (145) 72.75 ± 1.54a | (53) 26.7 ± 2.13a |
| IVM_O1 | 10^-9^ | 200 | (159) 79.75 ± 1.65b | (68) 34.0 ± 2.27b |
| IVM_O2 | 10^-8^ | 200 | (160) 80.0 ± 3.48b | (69) 34.5 ± 2.78b |
| IVM_O3 | 10^-7^ | 200 | (143) 71.5 ± 1.32a | (55) 27.7 ± 2.13a |

21- **Melatonin enhances in vitro developmental competence of cumulus-oocyte complexes collected by ovum pick-up in prepubertal and adult dairy cattle**

**Juan Carlos Gutiérrez-Añez , 2021**

Ovum pick up method, IVM, means ± SEM

|  | Melatonin concentration  IVM | N of zygotes | Cleavage rate | 1-Blastocyst (early,nonexpand) rates D8 | 2-Advanced (expand,hatchinh,hatched) blastocyst rates | (1+2)Total blastocysts |
| --- | --- | --- | --- | --- | --- | --- |
|  | 0 | 201 | (135) 67.4±3.9 | (27) 13.6±2.4 %b | (16) 8.0±2.2 %b | 43b |
| IVM_P1 | 0.01 nM | 209 | (146) 69.9±2.6 | (50) 24.0±3%a | (28) 13.2±2.1 %a | 78a |

|  | N of expanded blastocysts | TE number | ICM number | Total number | ICM: total number ratio | ICM: TE ratio |
| --- | --- | --- | --- | --- | --- | --- |
| 0 | 19 | 53.9±3.8 | 27.3±2.9b | 81.2±5.8b | 0.34±0.01b | 0.52±0.04a |
| 0.01 nM | 21 | 58.5±3.7 | 39.1±2.8a | 98.6±5.7a | 0.39±0.01a | 0.69±0.04 b |

22- **Melatonin improves the fertilization capacity and developmental ability of bovine oocytes by regulating cytoplasmic maturation events**

**Xue-Ming Zhao, 2018**

**IVM**, means ± standard error

|  | Melatonin concentration  IVM | nuclear maturation rate n | ROS level | normal distribution rate of mitochondria | distribution rates of CGs | normal distribution rate of ER |
| --- | --- | --- | --- | --- | --- | --- |
|  | 0 | 73.9%, 2846/3852a | 1.6 cps a | 61.0% b | 61.0% a | 65.2% b |
| IVM_Q1 | 10^-7^ | 82.7%, 2826/3419 a | 0.9 cps c | 73.9% b | 79.8% b | 81.1% b |
| IVM_Q2 | 10^-9^ | 90.9%, 2936/3229b | 1.1 cps b | 88.2% a | 87.0% c | 93.6% a |
| IVM_Q3 | 10^-11^ | 86.6%, 2962/3421 b | 1.3 cps b | 76.7% b | 70.6% b | 79.6% b |

| concentration of melatonin | No. of MII oocytes | polyspermy oocytes | No. of two-pronuclear embryos | No. of unfertilized  oocytes |
| --- | --- | --- | --- | --- |
| 0 | 101 | 11 (10.9 ± 1.5%)a | 62 (61.4 ± 3.6%)c | 28 (27.7 ± 1.5%)a |
| 10-7 | 109 | 3 (2.8 ± 0.2%)b | 84 (77.1 ± 1.9%)b | 22 (20.2 ± 2.0%)b |
| 10-9 | 92 | 0 (0.0%)c | 86 (93.5 ± 3.4%)a | 6 (6.5 ± 0.1%)d |
| 10-11 | 135 | 3 (2.2 ± 0.1%)b | 109 (80.7 ±1.8%)b | 23 (17.0 ± 1.9%)c |

Treatment reduced the level of **ROS** in oocytes.

Treatment increased **GSH** in oocytes

Treatment improved the **distribution of cortical granules**.

10-9 group improved **ATP content**, the **distribution of mitochondria** and **ER** in oocytes.

The normal distribution rates of IP3R1 were significantly higher in 10–7 M and 10–9 M groups.

The CD9 protein expression was significantly higher in the three melatonin-treated groups than in the control group.

the promoter methylation level of CD9 of 10-9 M melatonin group was significantly lower than those of 10-11 M, 10-7 M and control groups.

The rates of **polyspermy** and **non-fertilization** reduced significantly with the treatment.

No. of two-pronuclear embryos improved significantly.

| concentration of melatonin | No. of MII oocytes (%) | No. of cleavage embryos (%) | No. of blastocysts (%) | Total cell number |
| --- | --- | --- | --- | --- |
| 0 | 268 | 198 (73.9 ± 5.6%)c | 69 (34.8 ± 2.8%)c | 96.6 ± 6.9 (n = 30)d |
| 10-7 | 274 | 228 (83.2 ± 6.7%)bc | 94 (41.2 ± 4.6%)b | 108.8 ± 8.0 (n = 33) b |
| 10-9 | 329 | 310 (94.2 ± 6.2%)a | 158 (51.0 ± 4.6%)a | 117.5 ± 9.1 (n = 35)a |
| 10-11 | 295 | 254 (86.1 ± 5.3%)b | 110 (43.1 ± 3.3%)b | 101.1 ± 8.9 (n = 32) c |

| Level of transcription of OS-relataed genes in oocytes | CAT↑, SOD1↑, GPX↑ |
| --- | --- |
| … in oocytes | Tet1↑, Tet2↑, Tet3↑, Dnmt1↓ |

23- **Beneficial Effect of Melatonin on Blastocyst In Vitro Production from Heat-Stressed Bovine Oocytes**

**Cebrian-Serrano, 2013**

|  | Melatonin  IVM | N | Cleavage rate  (n) | Blast rate | Number of blastocyst cells  (Mean± SEM) |
| --- | --- | --- | --- | --- | --- |
|  | 0 | 287 | (208)72.52 % | (88) 30.75% | 103.17 ± 3.67 |
| IVM_R1 | 10^-12^M | 287 | (222) 77.47% | (96) 33.28% | 103.25 ± 3.73 |
| IVM_R2 | 10^-9^M | 287 | (225) 78.57% | (103) 35.77%a | 99.44 ± 3.54 |
| IVM_R3 | 10^-4^M | 287 | (225) 78.57% | (74) 25.7%b | 104.01 ± 4.30 |
|  |  |  |  |  |  |
|  | 0 | 243 | (193) 79.24% | (7) 3.00%a | 97.7±5.35 |
| IVM_R4 | 10^-3^M | 243 | (186) 76.41% | (68) 28.02%b | 85.49±9.92 |

24-**Beneficial effects of melatonin on bovine oocytes maturation: a mechanistic approach**

**Tian, 2014**

**IVM, mean**  ±  **S.E.M**

|  | Melatonin concentration | Number of COCs | Cleavage rate (n) | Blastocyst rate | Cell number per blastocysts |
| --- | --- | --- | --- | --- | --- |
|  | 0 | 280 | (187) 66.9 ± 1.88% c | (74) 26.5 ± 3.26%bc | 76.7 ± 2.43b |
| IVM_S1 | 10-3 | 280 | (148) 53.01±2.84d | (56) 19.91±2.52c | 75.98±3.43b |
| IVM_S2 | 10-5 | 280 | (181) 64.74±2.52c | (98) 35.12±3.79ab | 85.29±2.94ab |
| IVM_S3 | 10-7 | 280 | (211) 75.51±3.16ab | (111) 39.57±2.52a | 92.15±3.92a |
| IVM_S4 | 10-9 | 280 | (224) 79.9 ± 1.93% a | (115) 41.2 ± 3.07%,a | 93.2 ± 3.32a |
| IVM_S5 | 10-11 | 280 | (193) 68.98±2.1bc | (98) 34.94±3.47ab | 85.78±3.92ab |

|  | Melatonin concentration | Number of oocytes | Cleavage rate | the blastocyst rate, | the hatched blastocyst rate | the mean cell number/blastocyst |
| --- | --- | --- | --- | --- | --- | --- |
|  | 0 | 181 | (143) 79.2 ± 1.6%a | (32) 17.5 ± 1.5%a | (10) 5.7 ± 0.8%a | 96.4 ± 6.3a |
| IVM_S6 | 10-9 M | 181 | (152) 84.1 ± 3.3%b | (55) 30.2 ± 1.9%b | (23) 12.7 ± 1.2%b | 124.4 ± 6.1b |

| Relative transcription of maturation key genes in oocytes | GDF9↑, MARF1↑, DNMT1a↑ |
| --- | --- |
| Relative transcription of expansion and development genes in cumulus cells | PTX3↑, HAS1↑, HAS2↑, LHR1↑, LHR2↑, EGFR↑ |

25-**Melatonin slightly alleviates the effect of heat shock on bovine oocytes and resulting blastocysts**

**Shira Yaacobi-Artz, 2020**

IVM, means ± SEM

|  | Melatonin concentration  IVM | Number of oocytes | Temperature | Cumulus expansion | Nuclear maturation  M2 |
| --- | --- | --- | --- | --- | --- |
|  | Ctrl | 398 | 38.5ºC | a | (252) 63.29±2.53%a |
| IVM_T1 | 1.0 x 10-4 M | 106 | 38.5ºC | bc | (64) 60.25±4.55a |
| IVM_T2 | 1.0 x 10-7 M | 207 | 38.5ºC | ab | (127) 61.26±3.04a |

Treatment with 10-4 M melatonin reduced the **expansion of cumulus cells** but 10-7 had no effect.

| Melatonin c. | N of oocytes | Cleavage at 44h postinsemination | Blastocyst /oocytes at D8 |
| --- | --- | --- | --- |
| 0 | 151 | (119) 78.94±5.67a | (25) 16.76±3.83a |
| 1.0 x 10-7 M | 198 | (148) 74.89±9.32a | (40) 20.26±4.21a |

26- **Melatonin supplementation during in vitro maturation of oocyte enhances subsequent development of bovine cloned embryos**

**Quanli An,2019**

**IVM, mean±SD**

|  | Melatonin concentration IVM | **Number of oocytes** | **Nuclear maturation rate**  **(n) mean±SD** |
| --- | --- | --- | --- |
|  | 0 | **276** | **(191) 69.26±2.93a** |
| IVM_U1 | 10−5 | **235** | **(159) 67.8±2.44a** |
| IVM_U2 | 10−7 | **246** | **(199) 80.97±1.46b** |
| IVM_U3 | 10−9 | **253** | **(217) 85.85±2.44b** |
| IVM_U4 | 10−11 M | **231** | **(175) 75.6±1.96ab** |

| Melatonin concentration IVM  (IVF-derived embryos) | **Number of oocytes** | **Cleavage rate**  **(n) mean±SD** | **Blastocyst rate**  **mean±SD** |
| --- | --- | --- | --- |
| 0 | **196** | **(140) 71.35±3.08a** | **(63) 32.27±3.04a** |
| 10−5 | **182** | **(134) 73.78±2.56a** | **(61) 33.47±1.77a** |
| 10−7 | **189** | **(167) 88.56±2.05b** | **(87) 45.86±1.78bc** |
| 10−9 | **169** | **(151) 89.49±4.1b** | **(81) 47.84±2.03c** |
| 10−11 M | **187** | **(142) 76.06±3.59ab** | **(67) 35.61±2.03ab** |

| Melatonin concentration IVM  **(cloned embryos)** | **Number of oocytes** | **Cleavage rate**  **mean±SD** | **Blastocyst rate**  **mean±SD** |
| --- | --- | --- | --- |
| 0 | **187** | **71.35±2.05a** | **27.56±1.26a** |
| 10−5 | **169** | **71.21±1.54a** | **30.01±1.77a** |
| 10−7 | **183** | **82.4±2.05b** | **40.07±1.01b** |
| 10−9 | **185** | **84.35±2.06b** | **40.53±1.77b** |
| 10−11 M | **151** | **72.98±2.05a** | **30.38±2.02a** |

The **total cell number**, **ICM cells number** and **ICM:TE ratio** increased in the cloned embryos of treatment group.

The **total cell number** and **TE cell numbers** were higher in blastocysts of the IVF‐M(10-9) group (The extraction of data from graphs was impossible). The ICM and ICM/TE ratios were not significant.

The proportion of **apoptic cells** (TUNEL+) in embryos reduced significantly with treatment in IVF and cloned embryos.

10−9 M melatonin prominently reduced the relative **ROS** levels, and **early apoptosis** percentage in the bovine oocytes.

Treatment improved the proportions of oocytes with **distributed homogeneously mitochondria (V-FITC)**.

the incidence of the **disorganized spindle** with misaligned chromosome was lower in the melatonin groups than in the control group (anti‐α‐tubulin FITC antibody+DAPI staining).

27- **Protective effects of melatonin on the in vitro developmental competence of bovine oocytes**

**PANG, 2018**

**IVM, means** ± **standard errors**

|  | Melatonin concentration | Number | Polar body extrusion rate (n) % ± SEM |
| --- | --- | --- | --- |
|  | 0 | 178 | (134) 74.8 ± 3.2b |
| IVM_V1 | 10-11 | 167 | (133) 79.1 ± 1.3a |
| IVM_V2 | 10-9 | 177 | (146) 82.5 ± 1.7a |
| IVM_V3 | 10-7 | 169 | (125) 73.6 ± 2.2b |

Melatonin supplementation significantly increased the number of cytoplasmically matured oocytes.

10-9 group increased the distribution of CGs, proportion of oocytes with **homogeneous distributed mitochondria**, and **MMP**.

Treatment reduced the level of **ROS**, and **early apoptosis** (AnnexinV), and increased **GSH** in oocytes.

**Apoptosis rate** (TUNEL+) in embryos decreased with the treatment.

| Melatonin concentration | Number of zygotes | No. of embryos cleaved (%± SEM) | No. of blastocysts (%± SEM) |
| --- | --- | --- | --- |
| 0 | 156 | 140 (89.8 ± 0.6)a | 51 (32.8 ± 0.5)c |
| 10-11 | 182 | 153 (84.6 ± 8.2)a | 70 (38.6 ± 1.2)b |
| 10-9 | 173 | 151 (87.3 ± 0.4)a | 73 (42.2 ± 0.8)a |
| 10-7 | 137 | 122 (89.6 ± 4.7)a | 51 (37.3 ± 0.5)b |

| Melatonin concentration | Number of embryos | Number of total cells | Apoptotic cells rate % |
| --- | --- | --- | --- |
| 0 | 51 | 76.08±9.8a | 7.45±1.24 a |
| 10-11 | 51 | 79.23±4.51a | 5.98±0.32b |
| 10-9 | 51 | 82.37±4.52a | 4.53±0.39 c |
| 10-7 | 51 | 79.06±9.36a | 5.87±0.72 bc |

| Relative expression of apoptosis-related genes in embryos (10^-9^ group) | Caspase↓, BAX, BCL2↑, XIAP↑ |
| --- | --- |
| Relative expression of antioxidant-related genes in embryos | CAT↑ |
| Relative expression of stress-related genes in embryos | HSP70↑ |

28-**Developmental, cytogenetic and epigenetic consequences of removing complex proteins and adding melatonin during in vitro maturation of bovine oocytes**

**Tutt, 2023**

IVM, OPU oocytes, means ± SEM

|  | M concentration | Number of COCs | Cleavage rate %(n) | Blastocysts at Day 6, % | Blastocysts/oocytes % at D8 | Hatched blastocysts at D8 |
| --- | --- | --- | --- | --- | --- | --- |
|  | 0 | 105 | (102) 97.5 ± 2.11a | 14.7 ± 7.98 | (64) 61.4 ± 6.68 | (43) 40.7 ± 7.78 |
| IVM_W1 | 100 nM | 105 | (92) 87.5 ± 5.67b | 31.5 ± 8.26 | (71) 67.5 ± 5.48 | (58) 55.1 ± 6.43 |

**29- Effects of EGF and melatonin on gene expression of cumulus cells and further in vitro embryo development in bovines**

**Cordova, 2022**

IVM, parthenogenesis

Nuclear maturation: 75.9% and non-significant.

|  | Melatonin concentration, IVM | Number of mature oocytes | Cleavage rate (n) Mean±SEM | Blastocyst rate |
| --- | --- | --- | --- | --- |
|  | 0 | 211 | (133) 63±0.68a | (42) 20±0.34a |
| IVM_X1 | 50 ng/ml (2.15*10^-4^M) | 200 | (160) 80±0.68b | (64) 32±0.34b |

| Relative transcription in CCs | TNFAIP6, GREM1, HAS2, PTX3, GPX1, CuZnSOD↓ |
| --- | --- |
| Relative transcription in blastocysts | BAX/BCL2 ratio, OCT4 |

**30-Melatonin improves the quality of frozen bull semen and influences gene expression related to embryo genome activation**

**Su, 2021**

IVM

|  | Melatonin concentration, IVM | Number of COCs | Maturation rate % |
| --- | --- | --- | --- |
|  | 0 | 443 | (318) 71.83%a |
| IVM_Y1 | 10-6 | 293 | (221) 75.38 |
| IVM_Y2 | 10-7 | 331 | (267) 80.73b |
| IVM_Y3 | 10-8 | 280 | (205) 73.2a |
| IVM_Y4 | 10-9 | 316 | (231) 73.06a |
| IVM_Y5 | 10-10 | 332 | (236) 71.06a |

**ROS** level significantly decreased in treatment group (10-7 M)
