# Supplementary material for: Effect of Melatonin Supplementation on In Vitro Developmental Competence of Bovine Oocyte: A Systematic Review and Meta-Analysis
Source: Vet Med Int. 2025 Oct 24;2025:5351950. doi: 10.1155/vmi/5351950 (PMC12578564; doi:10.1155/vmi/5351950)
Supplement: Supporting Information 5 — Supporting 5: Abbreviation table. [file 5351950.f5.doc]

Abbreviation table

| **Abbreviation** | **Explanation** |
| --- | --- |
| IVEP | In Vitro Embryo Production |
| OR | Odds Ratio |
| CI | Confidence Interval |
| ROS | Reactive Oxygen Species |
| OS | Oxidative Stress |
| COCs | Cumulus Oocyte Complexes |
| PRISMA | Preferred Reporting Items for Systematic Reviews and Meta-Analyses |
| WOS | Web of Sciences |
| P.I.C.O | Population. Intervention. Control. Outcome |
| OPU | Ovum Pick Up |
| I^2^ | I-squared |
| NM | Nuclear Maturation |
| CR | Cleavage Rate |
| BR | Blastocyst Rate |
| HBR | Hatched-Blastocyst Rate |
| CCs | Cumulus Cells |
| ER | Endoplasmic Reticulum |
| MMP | Mitochondrial Membrane Potential |
| ATP | Adenosine triphosphate |
| GSH | Glutathione |
| CAT | Catalase |
| SOD | Superoxide dismutase |
| GPX | Glutathione peroxidase |
| COMET | a single cell gel electrophoresis assay |
| TCN | Total Cell Number |
| ICM | Inner Cell Mass |
| TE | Trophectoderm |
| CYP11A | Cytochrome P450 family 11 subfamily A |
| StAR | Steroidogenic Acute Regulatory Protein |
| PTX | Paclitaxel |
| HAS | Hyaluronan Synthase |
| LHR | Luteinizing Hormone Receptor |
| EGFR | Epidermal Growth Factor Receptor |
| TNFAIP | Tumor Necrosis Factor Alpha-induced Protein |
| GREM1 | Gremlin 1 |
| Cu,ZnSOD | Copper-Zinc Superoxide Dismutase (also known as SOD1) |
| MnSOD, | Manganese Superoxide Dismutase |
| Tet1 | Ten-eleven Translocation Methylcytosine Dioxygenase |
| GDF9 | Growth Differentiation Factor 9 |
| MARF1 | Meiosis Regulator And mRNA Stability Factor 1 |
| DNMT | DNA (cytosine-5)-Methyltransferase |
| XIAP | X-linked Inhibitor of Apoptosis Protein |
| MCL | Myeloid Cell Leukemia |
| BCL-2 | B-cell Lymphoma 2 |
| CASP | Caspase |
| BAX | Bcl-2-Associated X protein |
| SHC-1 | SHC-transforming protein 1 (Shc family of adaptor proteins) |
| HSP | Heat Shock Protein |
| OCT4, | Octamer-binding transcription factor 4 (also known as POU5F1) |
| NANOG | hNanog (Tir Na Nog) |
| PU5F1 | POU class 5 homeobox 1 |
| SLC | Solute Carrier Family (known as neutral amino acid transporters) |
| HSPB1 | Heat Shock Protein Beta-1 (also known as Hsp27) |
| KRT8 | Keratin 8 |
| ART | Assisted Reproductive Techniques |
| CGs | Cortical Granuls |
| IVC | In Vitro Culture |
